# Ethnopharmacological insights into type 2 diabetes management by traditional Moroccan herbal teas: HPLC-MS characterization of phenolic compounds and their related antioxidant and enzyme inhibitory activities

**DOI:** 10.1186/s12906-026-05588-9

**Published:** 2026-09-26

**Authors:** Ahmed Zayed, Meryem El Jemli, Mourad Kharbach, My El Abbes Faouzi, Rasha Ali Radwan, Shahira M. Ezzat

**Affiliations:** 1 Department of Pharmacognosy, College of Pharmacy, Tanta University, El Guish Street, Tanta, 31527 Egypt; 2 Research Laboratory in Drug Sciences, Mohammed VI Faculty of Pharmacy, Mohammed VI University of Sciences and Health, Casablanca, Morocco; 3 Research Unit, Mohammed VI Center for Research and Innovation, Rabat, Morocco; 4 Circular Economy/Sustainable Material Solutions, Faculty of Technology, LAB University of Applied Sciences, Mukkulankatu 19, Lahti, 15101 Finland; 5 Laboratories of Pharmacology and Toxicology, Faculty of Medicine and Pharmacy, Mohammed V University in Rabat, Rabat, Morocco; 6 Biochemistry Department, Faculty of Biotechnology, German International University (GIU), Regional Ring Road, New Administrative Capital , East Cairo Egypt; 7 Department of Pharmacognosy, Faculty of Pharmacy, Cairo University, Kasr El-Aini Street, Cairo, 11562 Egypt; 8 Department of Pharmacognosy, Faculty of Pharmacy, October University for Modern Sciences and Arts (MSA), Giza, 12451 Egypt

**Keywords:** Antioxidant, Diabetes mellitus, Enzyme inhibition, Herbal remedies, Phytochemicals

## Abstract

**Background:**

Traditional Moroccan herbal teas are widely consumed and have been traditionally used for managing chronic metabolic disorders, particularly type 2 diabetes mellitus (T2DM). Although these preparations have a long history of ethnomedicinal use, their phenolic composition and biological activities remain insufficiently characterized. Scientific validation is needed to support their traditional application and provide evidence for their potential relevance to T2DM management. This study aimed to characterize the phenolic profile, antioxidant potential, and carbohydrate-digestive enzyme inhibitory activity of 21 traditional Moroccan herbal teas used for glycaemic control.

**Methods:**

Phenolic constituents were identified and quantified using HPLC-MS. Antioxidant capacity was evaluated through DPPH, ABTS, and FRAP assays. Inhibitory effects on *α*-amylase and *α*-glucosidase were assessed in vitro. Correlation analyses were conducted to explore associations between phenolic constituents and biological activities.

**Findings:**

Marked variability in total phenolic content (TPC) was observed, with *Mentha × piperita* and *Thymus broussonetii* exhibiting the highest levels at 883.2 ± 13.0 and 746.8 ± 22.4 mg GAE/g dry weight, respectively. Antioxidant activity varied substantially among the investigated teas, with *T. broussonetii* (FRAP reducing power: 423.6 ± 9.8 µg/mL) and *Mentha rotundifolia* (162.6 ± 0.6 µg/mL) showing significant reducing capacities. TPC exhibited strong positive correlations with antioxidant indices (*r* = 0.93–0.96). *Pelargonium graveolens* presented potent *α*-amylase inhibition (IC_50_ = 2.98 ± 1.7 µg/mL), whereas *Mentha viridis* and *Calamintha officinalis* were among the most active *α*-glucosidase inhibitors (IC_50_ = 1.9 ± 0.4 and 1.2 ± 0.1 µg/mL, respectively). Negative correlations were found between rutin (*r* = − 0.49) and gallic acid (*r* = − 0.44) contents and *α*-amylase IC_50_ values.

**Conclusion:**

The investigated Moroccan herbal teas exhibited variable antioxidant and digestive-enzyme inhibitory activities that were associated with their phenolic composition. Variability among samples may reflect differences in plant species and environmental or agronomic factors. These findings provide comparative in vitro evidence supporting the traditional use of Moroccan herbal teas and highlight several species as promising sources of bioactive phenolics. However, the present findings do not establish therapeutic efficacy, and further in vivo, mechanistic, pharmacokinetic, and clinical studies are required to confirm their efficacy, safety, and clinical relevance.

**Supplementary Information:**

The online version contains supplementary material available at https://doi.org/10.1186/s12906-026-05588-9.

## Introduction

Diabetes mellitus (DM) is a chronic metabolic disorder characterized by persistent hyperglycemia, defined as fasting plasma glucose ≥ 126 mg/dL or random plasma glucose ≥ 200 mg/dL [1]. It results from impaired insulin secretion, insulin resistance, or both, leading to metabolic disturbances that cause progressive dysfunction of vital organs such as the eyes, kidneys, nerves, heart, and blood vessels [2]. According to the International Diabetes Federation (IDF), 11th Edition, an estimated 589 million adults aged 20–79 years, representing approximately 11.1% of the global adult population, are currently living with diabetes, and this number is projected to rise to 853 million by 2050. Type 2 diabetes mellitus (T2DM) accounts for more than 95% of all cases worldwide. In Morocco, the estimated prevalence of diabetes among adults (20–79 years) is 11.9%, equivalent to approximately 2.9 million people living with the disease in 2024, underscoring a major national health challenge [3]. The growing burden of T2DM in Morocco is strongly associated with urbanization, changes in dietary habits, and increasingly sedentary lifestyles, all of which have contributed to a decline in the use of traditional herbal therapies once deeply rooted in Moroccan culture [4].

At the biochemical level, oxidative stress plays a central role in the development and progression of T2DM. Excessive production of reactive oxygen species (ROS) contributes to insulin resistance, *β*-cell dysfunction, and glucose intolerance [5]. Natural phenolic compounds have been reported to exert multifaceted antidiabetic effects through ROS scavenging, enhancement of endogenous antioxidant defenses, modulation of glucose uptake, and protection of pancreatic *β*-cells [6, 7]. In parallel, inhibition of carbohydrate-digesting enzymes such as *α*-amylase and *α*-glucosidase represents a validated therapeutic strategy for managing postprandial hyperglycemia [8–10]. These enzymes catalyze the hydrolysis of complex carbohydrates into glucose, and their inhibition delays intestinal glucose absorption, thereby attenuating blood sugar spikes. Furthermore, plant-derived phenolic compounds may mitigate diabetic complications by reducing the formation of advanced glycation end-products (AGEs), which are implicated in vascular and neuropathic damage [6, 11, 12].

In Morocco, herbal remedies remain an important component of traditional healthcare, particularly in rural communities, where herbal teas are widely consumed for promoting metabolic health and managing diabetes. Ethnobotanical surveys have documented the frequent use of numerous indigenous species, including *Thymus broussonetii* Boiss., *Mentha viridis* L., *Mentha × piperita* L., *Calamintha officinalis* Moench, *Pelargonium graveolens* L’Hér., *Origanum majorana* L., *Salvia officinalis* L., *Myrtus communis* L., and *Citrus aurantium* L., as traditional antidiabetic remedies [13–15]. Despite their widespread use, the scientific validation of these teas remains limited, and comprehensive comparisons among the various formulations traditionally used for glycemic control are largely absent from literature. Previous studies have predominantly focused on individual plants or extracts, with limited comparative assessment of their polyphenolic diversity and biological activities under standardized experimental conditions [16–19].

Moreover, recent pharmacological research has reinforced the potential of phenolic-rich plants as multifunctional agents capable of simultaneously inhibiting *α*-amylase and *α*-glucosidase while exerting antioxidant effects [20–22]. Advances in phytochemistry and pharmacology have also improved understanding of the bioavailability and pharmacokinetic properties of these compounds [23–25]. Furthermore, contemporary investigations highlight food-derived phenolic matrices, such as apple peel extracts, as promising agents against hyperglycemia and glycation stress [26], further demonstrating the continuing biomedical relevance of phenolic compounds in diabetes-related research.

Although numerous Moroccan medicinal plants have individually been investigated for their phytochemical composition and biological activities, comprehensive comparative studies evaluating a broad panel of traditionally consumed Moroccan herbal teas using a unified analytical workflow remain scarce. Specifically, few studies have simultaneously integrated phytochemical profiling with antioxidant and carbohydrate-digestive enzyme inhibition assays across multiple Moroccan herbal teas prepared under comparable conditions. To the best of our knowledge, this is among the first studies to comparatively investigate 21 Moroccan herbal teas by integrating targeted high-performance liquid chromatography coupled with mass spectrometry (HPLC-MS)-based phenolic profiling with antioxidant evaluation and dual *α*-amylase and *α*-glucosidase inhibitory assays under identical experimental conditions. This integrated comparative strategy represents the principal novelty of the present work and provides a more comprehensive assessment of the relationship between phenolic composition and biological activity than studies focusing on individual plants. Therefore, the present study aimed to characterize the phenolic composition of 21 Moroccan herbal teas using HPLC-MS, evaluate their antioxidant capacity using the 1,1-diphenyl-2-picrylhydrazyl (DPPH) radical scavenging assay, the 2,2′-azinobis(3-ethylbenzothiazoline-6-sulfonic acid) (ABTS•⁺) radical cation scavenging assay, and the ferric reducing antioxidant power (FRAP) assay, and determine their in vitro inhibitory activities against *α*-amylase and *α*-glucosidase. The generated data provide comparative scientific evidence supporting the traditional use of Moroccan herbal teas and identify promising candidates for further pharmacological investigation and potential development as standardized functional beverages or complementary dietary approaches for diabetes management.

## Methods

### Plants collection and identification

Twenty-one plants were selected for the present study, which are grouped in Table 1. The plants were chosen on the basis of ethnobotanical surveys, literature reports, and information obtained from local herbalists. These species are among the most commonly used ingredients in Moroccan herbal teas (“atay bel aâchab”), traditionally consumed for their beneficial effects on metabolic regulation, particularly in diabetes and obesity.

**Table 1:** Ethnobotanical information, collection localities, environmental conditions, voucher specimen numbers, and applied codes of the medicinal plants investigated in the current study

Specimen No.	Vernacular name	Scientific name	Family	Origin of plants	Environmental conditions	Voucher specimen No.	Applied codes
1	Arq souss	*Glycyrrhiza glabra* L.	Fabaceae	India	---*	LPT-FMP-RAB-001	GG
2	Attercha	*Pelargonium graveolens* L’Hér.	Geraniaceae	Mzoura (32°49’41.8"N, 7°51’34.5"W)	Temperature: 25 °C humidity 75% Altitude: 700 m	LPT-FMP-RAB-002	PG
3	Azir	*Rosmarinus officinalis* L.	Lamiaceae	Mzoura (32°49’41.8"N, 7°51’34.5"W)	Temperature: 25 °C humidity 75% Altitude: 700 m	LPT-FMP-RAB-003	RO
4	Badina	*Illicium verum* Hook.f.	Schisandraceae	China	---	LPT-FMP-RAB-004	BD
5	Babounj	*Matricaria chamomilla* L.	Asteraceae	Mzoura (32°49’41.8"N, 7°51’34.5"W)	Temperature: 25 °C humidity 75% Altitude: 700 m	LPT-FMP-RAB-005	MC
6	Chiba	*Artemisia absinthium* L.	Asteraceae	Mzoura (32°49’41.8"N, 7°51’34.5"W)	Temperature: 25 °C humidity 75% Altitude: 700 m	LPT-FMP-RAB-006	AA
7	Flio	*Mentha pulegium* L.	Lamiaceae	Mzoura (32°49’41.8"N, 7°51’34.5"W)	Temperature: 25 °C humidity 75% Altitude: 700 m	LPT-FMP-RAB-007	MP
8	Kamoun soufi	*Ammodaucus leucotrichus* Coss. & Dur.	Apiaceae	Zagora (30°20’34.4"N, 5°50’16.1"W)	Temperature: 38 °C humidity 44% Altitude: 870 m	LPT-FMP-RAB-008	AL
9	Karkadi	*Hibiscus sabdariffa* L.	Malvaceae	India	---	LPT-FMP-RAB-009	HS
10	Lwiza	*Lippia citriodora* Kunth.	Verbenaceae	Mzoura (32°49’41.8"N, 7°51’34.5"W)	Temperature: 25 °C humidity 75% Altitude: 700 m	LPT-FMP-RAB-010	LC
11	Mantha	*Calamintha officinalis* Moench.	Lamiaceae	Mzoura (32°49’41.8"N, 7°51’34.5"W)	Temperature: 25 °C humidity 75% Altitude: 700 m	LPT-FMP-RAB-011	CO
12	Merdedouch	*Origanum majorana* L.	Lamiaceae	Mzoura (32°49’41.8"N, 7°51’34.5"W)	Temperature: 25 °C humidity 75% Altitude: 700 m	LPT-FMP-RAB-012	OM
13	Naanaa	*Mentha viridis* L.	Lamiaceae	Mzoura (32°49’41.8"N, 7°51’34.5"W)	Temperature: 25 °C humidity 75% Altitude: 700 m	LPT-FMP-RAB-013	MV
14	Naanaa abdi	*Mentha* x *piperita* L.	Lamiaceae	Mzoura (32°49’41.8"N, 7°51’34.5"W)	Temperature: 25 °C humidity 75% Altitude: 700 m	LPT-FMP-RAB-014	NA
15	Nafaa	*Foeniculum vulgare* Mill.	Apiaceae	Ourika (31°23’26.0"N 7°50’11.0"W)	Temperature: 30 °C humidity 45% Altitude: 1300 m	LPT-FMP-RAB-015	FV
16	Rihan	*Myrtus communis* L.	Myrtaceae	Ourika (31°23’26.0"N 7°50’11.0"W)	Temperature: 30 °C humidity 45% Altitude: 1300 m	LPT-FMP-RAB-016	RI
17	Salmia	*Salvia officinalis* L.	Lamiaceae	Mzoura (32°49’41.8"N, 7°51’34.5"W)	Temperature: 25 °C humidity 75% Altitude: 700 m	LPT-FMP-RAB-017	SO
18	Timija/Marsita	*Mentha rotundifolia* L.	Lamiaceae	Mzoura (32°49’41.8"N, 7°51’34.5"W)	Temperature: 25 °C humidity 75% Altitude: 700 m	LPT-FMP-RAB-018	MR
19	Zaatar	*Origanum compactum* Benth.	Lamiaceae	Ourika (31°23’26.0"N 7°50’11.0"W)	Temperature: 30 °C humidity 45% Altitude: 1300 m	LPT-FMP-RAB-019	OC
20	Zaaietra	*Thymus broussonetti* Boiss.	Lamiaceae	Ourika (31°23’26.0"N 7°50’11.0"W)	Temperature: 30 °C humidity 45% Altitude: 1300 m	LPT-FMP-RAB-020	TB
21	Zhar Lrnej	*Citrus aurantium* L.	Rutaceae	Mzoura (32°49’41.8"N, 7°51’34.5"W)	Temperature: 25 °C humidity 75% Altitude: 700 m	LPT-FMP-RAB-021	CA

---*: Not available

The plant materials were obtained from several sources. Most species were directly collected from organic farms located in the Mzoura, Ourika, and Zagora regions of Morocco, while a minority of species originating from China or India were obtained through certified herbalists because they are not naturally cultivated in Morocco. All plants were harvested during their optimal growing seasons (March-June) to ensure high phytochemical content. The precise collection localities, GPS coordinates, environmental conditions, and voucher specimen numbers are provided in Table 1.

Botanical identification was carried out by Dr. Ilias Marmouzi (Faculty of Medicine and Pharmacy, Mohammed V University, Rabat, Morocco) using standard floristic references, including *Catalogue des plantes vasculaires du Maroc* and *Flore pratique du Maroc*. Voucher specimens for all investigated plants were prepared, authenticated, assigned unique accession numbers according to the Herbarium coding system of the Laboratory of Pharmacology and Toxicology (LPT), Faculty of Medicine and Pharmacy (FMP), Mohammed V University, Rabat (RAB), and deposited in the Herbarium of the Laboratory of Pharmacology and Toxicology, Faculty of Medicine and Pharmacy, Mohammed V University, Rabat, Morocco. Voucher specimen information, including accession numbers, vernacular names, scientific names, plant families, collection localities, and applied abbreviation codes, is presented in Table 1.

All experimental procedures were conducted in accordance with relevant institutional, national, and international guidelines and regulations. Appropriate permissions were obtained for the collection of plant materials and preparation of voucher specimens.

### Plants extraction and sample preparation for analysis

The collected plant materials were thoroughly cleaned, air-dried in the shade at ambient temperature (25.0 ± 2.0 °C) to preserve their phytochemical integrity, and then ground into fine powders using a mechanical grinder. For the extraction process, 30 g of each powdered sample was infused with 300 mL of boiling distilled water 100 °C (1:10 *w/v*) for 30 min using the hot-infusion method, ensuring efficient extraction of water-soluble phytoconstituents. After 30 min at room temperature, the mixtures were filtered through Whatman No. 1 filter paper to remove plant residues.

The filtrates were concentrated under reduced pressure at 40 °C using a rotary evaporator and subsequently lyophilized. Lyophilization was performed by freezing the extract at -80 °C for 24 h using a Freeze Dry System (FreeZone Benchtop, Labconco, Kansas City, MO, USA) under vacuum for 48 h. The obtained lyophilized extracts were weighed to calculate the extraction yield and stored in airtight amber containers at 4 °C until further analysis. All extractions were performed in triplicate (*n* = 3) to ensure reproducibility and minimize experimental variability [27].

This method was chosen as it reflects the traditional Moroccan practice of preparing herbal tea (“atay bel aâchab”), allowing for efficient extraction of water-soluble phytochemicals commonly used in local medicine [28].

### Phytochemical analysis

#### Determination of total phenolic content in investigated Moroccan herbal extracts

The total phenolic content (TPC) of the plant extracts was determined using the Folin-Ciocalteu colorimetric method, as described by Lister and Wilson, with minor modifications. This method is based on the reduction of the Folin-Ciocalteu reagent by phenolic compounds under alkaline conditions, resulting in the formation of a blue-colored complex that can be measured spectrophotometrically [29]. Briefly, an aliquot of 25 µL of each plant extract, prepared by dissolving 1 mg of dry extract in 1 mL of distilled water, was mixed with 125 µL of 10% (*v/v*) Folin-Ciocalteu reagent. After 5 min of incubation at room temperature, 100 µL of 7.5% (*w/v*) sodium carbonate (Na_2_CO_3_) solution was added to the mixture. The reaction mixture was vortexed and incubated in the dark at room temperature for 30 min to allow for complete color development.

The absorbance of the resulting blue color was measured at 765 nm using a UV-Vis spectrophotometer plate reader (ELX 808, Bio-Tek Instrumental, Italy) against a reagent blank prepared similarly but without plant extract. A standard calibration curve was constructed using gallic acid solutions (concentration range: 0–200 µg/mL). The TPC of the extracts was expressed as mg of gallic acid equivalents/g of dry weight (dw) of extract (mg GAE/g dw). All determinations were performed in triplicate, and results were expressed as mean ± standard deviation (SD).

#### Quantification of phenolic compounds by HPLC-MS

The definitive quantification of the target phenolic compounds was executed using an optimized HPLC-MS procedure. This method, adapted with critical modifications from [30], was systematically tailored to maximize accuracy, selectivity, and reproducibility for the complex matrix of Moroccan herbal tea extracts.

Analyses were performed on an advanced HPLC system hyphenated with a Diode-Array Detector (DAD) and an Electrospray Ionization (ESI) Mass Spectrometer. The ESI source was judiciously operated in the negative ion mode, a setting specifically optimized for the highly sensitive ionization of a broad range of phenolic acids and flavonoids, which readily deprotonate ([M–H]^–^) under these conditions.

Chromatographic separation was achieved using a C_18_ reversed-phase column (250 mm × 4.6 mm, 5 μm particle size). Maintaining the column temperature at a precise 25 °C was critical to ensure constant retention times and mitigate the variability associated with temperature-sensitive chromatographic phases. The mobile phase utilized an aqueous component, Solvent A (0.1% formic acid in water), and an organic component, Solvent B (acetonitrile). The addition of 0.1% formic acid in the aqueous phase serves a dual purpose; it acts as a volatile mobile phase additive that promotes efficient ESI by providing protons and ensures robust peak shape by suppressing the ionization of acidic analytes in the solvent. The gradient elution program was meticulously developed to achieve optimal chromatographic separation of compounds exhibiting diverse polarities. The analysis commenced with an isocratic hold at 5% Solvent B (acetonitrile) for the initial 5 min, allowing for effective equilibration of the column and robust separation of highly polar compounds, such as simple phenolic acids. The organic phase was then gradually increased from 5% to 25% B over the next 20 min (5–25 min), facilitating the elution of medium-polarity phenolic acids and glycosylated flavonoids. A subsequent ramp to 50% B over 10 min (25–35 min) was implemented to elute less polar flavonoid glycosides and intermediate metabolites. Following this, a rapid increase to a high organic percentage (95% B) over 10 min (35–45 min) was necessary to effectively strip highly non-polar compounds, such as lipophilic aglycones, from the stationary phase. This was followed by a 5-min isocratic wash at 95% B to ensure the column was fully cleaned of strongly retained species, after which the system was re-equilibrated to the initial conditions for subsequent injections, ensuring baseline stability and reliable retention times. The consistent flow rate of 0.5 mL.min^− 1^ and a precise injection volume of 10 µL were maintained for all injections to ensure comparative peak areas and minimal column overloading.

A comprehensive panel of authenticated phenolic reference standards, including gallic acid, catechin, chlorogenic acid, caffeic acid, rutin, quercetin, rosmarinic acid, and ferulic acid, was used for unequivocal identification and accurate quantification. Stock solutions (1 mg mL^− 1^) were prepared in HPLC-grade methanol and serially diluted to produce working standards spanning 0.1–100 µg mL^− 1^, the calibration range selected to encompass the expected analyte concentrations in the extracts.

Lyophilized crude extracts were accurately weighed, reconstituted in methanol at 10 mg mL^− 1^, and filtered through 0.22 μm PTFE syringe filters to remove particulate matter prior to HPLC-MS injection and analysis.

Compound identification was achieved by comparing the chromatographic and mass spectrometric characteristics of each detected peak with those of authenticated reference standards analyzed under identical chromatographic conditions. Identification was based on the agreement of retention time (RT), molecular weight, the deprotonated molecular ion ([M − H]⁻) detected in negative electrospray ionization mode, and characteristic MS fragmentation patterns. Compound assignments were further verified by comparison with published literature data and validated spectral libraries whenever applicable.

Quantification was performed using external calibration curves constructed from authentic reference standards. Calibration curves were established over the concentration range of 0.1–100 µg/mL and demonstrated excellent linearity for all investigated compounds (R^2^ = 0.9938–0.9999), as summarized in Suppl. Table S1. Dual-wavelength DAD detection was performed at 280 nm for general phenolic compounds and at 320 nm for hydroxycinnamic acids, including chlorogenic, caffeic, ferulic, and rosmarinic acids, to improve the selectivity of chromatographic detection. Quantitative results are expressed as mg/kg dry weight (dw). All analyses were performed in triplicate (*n* = 3), and the results are presented as mean ± standard deviation (SD).

#### Biological activities of investigated Moroccan herbal teas

Prior to biological activity investigations, i.e., antioxidant and enzyme inhibition assays, lyophilized extracts were reconstituted in methanol: water (80:20, *v/v*) at 10 mg mL^− 1^, vortexed for 1 min, sonicated for 10 min, centrifuged at 10,000 × *g* for 10 min, and filtered through 0.22 μm PTFE filters.

#### Antioxidant activity

The antioxidant capacity of the Moroccan herbal tea extracts was assessed using three complementary in vitro assays: DPPH, ABTS, and FRAP. All assays included appropriate positive and negative controls. Antioxidant activity was evaluated from concentration-response experiments, and the results are presented as IC_50_ values, corresponding to the extract concentration required to produce 50% radical scavenging activity (DPPH and ABTS) or 50% ferric reducing activity (FRAP) [31, 32].

#### DPPH radical scavenging activity

Free radical scavenging activity was evaluated using the DPPH assay as described by [33], with modifications. Briefly, 100 µL of extract solution at varying concentrations (7.81–1000 µg/mL) was mixed with 3.9 mL of 0.1 mM DPPH methanolic solution. The mixture was incubated in the dark at room temperature for 30 min, and absorbance was recorded at 517 nm using a UV-Vis spectrophotometer. The scavenging effect was calculated using Eq. 1:

1 $$\begin{aligned}\mathrm{DPPH}&\:\mathrm{Radical}\:\mathrm{scavenging}\left(\%\right)=\\&\left[\left(\text{A control}-\text{A sample}\right)/\text{A control}\right]\\&\times100\end{aligned}$$

Trolox was used as a positive control, and a blank without extract served as the negative control. IC_50_ values in µg/mL were calculated from the concentration-response curves generated for each extract.

#### ABTS radical cation scavenging activity

The ABTS assay was performed following [34]. ABTS⁺ radicals were generated by reacting 7 mM ABTS with 2.45 mM potassium persulfate for 16 h in the dark. The solution was diluted with ethanol to an absorbance of 0.70 ± 0.02 at 734 nm. Then, 100 µL of extract solution was mixed with 3.9 mL of ABTS⁺ solution and incubated for 10 min at room temperature. Absorbance was measured at 734 nm, and radical scavenging activity was calculated as demonstrated in Eq. 2:

2 $$\begin{aligned}\mathrm{ABTS}&\:\mathrm{scavenging}\left(\%\right)=\\&\left[\left(\text{A blank}--\text{A sample}\right)/\text{A blank}\right]\\&\times100\end{aligned}$$

Trolox was used as positive control, and a blank without extract as a negative control. IC_50_ values were determined from the corresponding concentration-response curves and are expressed as µg/mL.

#### FRAP reducing power assay

The FRAP assay was conducted following [35], with modifications. The assay measures the reduction of Fe^3+^ to Fe^2+^ in the presence of antioxidants. Briefly, 1 mL of extract was mixed with 2.5 mL of 0.2 M phosphate buffer (pH 6.6) and 2.5 mL of 1% potassium ferricyanide, then incubated at 50 °C for 20 min. After incubation, 2.5 mL of 10% trichloroacetic acid was added, and the mixture was centrifuged at 3000 rpm for 10 min. Then, 2.5 mL of supernatant was combined with 2.5 mL of distilled water and 0.5 mL of 0.1% FeCl_3_ solution. Absorbance was measured at 700 nm.

Similar to Trolox previously used in ABTS assay, other standard compounds such as ascorbic acid, catechin, and gallic acid are commonly employed in antioxidant assays for comparison [36, 37]. Ascorbic acid was used as a positive control, and a blank without extract served as a negative control.

#### Alpha-amylase inhibition

The *α*-amylase inhibitory assay was performed according to [38]. Preliminary screening experiments were initially conducted to establish the appropriate concentration range for each extract. The (3.4–17.2) µg/mL range was subsequently used for enzyme inhibition assays. A reaction mixture without inhibitor served as the negative control. Each extract solution was mixed with soluble starch (substrate) and *α*-amylase enzyme under alkaline conditions at 25 °C. Maltose production was assessed by the reduction of 3,5-dinitrosalicylic acid (DNS) to 3-amino-5-nitrosalicylic acid, with absorbance measured at 540 nm. Inhibition (%) was calculated as using Eq. 3 as previously described in literature [39, 40].

3 $$\begin{aligned}\alpha-&\text{Amylase inhibition}\left(\%\right)=\\&\left[\left(\mathrm{A}\_\mathrm{control}-\mathrm{A}\_\mathrm{sample}\right)/\mathrm{A}\_\mathrm{control}\right]\\&\times100\end{aligned}$$

#### Alpha-glucosidase inhibition

The *α*-glucosidase inhibitory assay followed [41]. Preliminary experiments were conducted to identify the concentration range encompassing for each extract. The (0.00166–0.83) µg/mL range was used for IC_50_ determination. In brief, 10 µL of phosphate buffer (pH 6.8) was mixed with 30 µL of *α*-glucosidase enzyme solution (0.017 units/mL) and 10 µL of extract. Mixtures were incubated at 37 °C for 15 min, followed by addition of 100 µL of 0.7 mM *p*-nitrophenyl-*α*-D-glucopyranoside (PNPG) substrate and further incubation at 37 °C for 30 min. Absorbance was read at 405 nm. The reaction mixture without inhibitor served as the negative control. Inhibition (%) was calculated using Eq. 4 [39, 40]:

4 $$\begin{aligned}\alpha-\mathrm{Glucosidase}&\:\mathrm{inhibition}\left(\%\right)=\\&\left[\left(\text{A control}--\text{A sample}\right)/\text{A control}\right]\\&\times100\end{aligned}$$

### Statistical analysis

All assays were performed in triplicate, and the results are expressed as mean ± standard deviation (SD). The IC_50_ values for each plant extract against *α*-amylase and *α*-glucosidase were estimated from 50 × concentration / % inhibition. The final IC_50_ reported is the arithmetic mean of the estimates obtained from the different tested concentrations of the same extract. This approach provides an approximate IC_50_ estimation but does not account for the complete concentration–response curve. Microsoft Excel 2010 was used for data processing and IC_50_ calculations. Before performing parametric analyses, data distribution was assessed for normality and homogeneity of variances. Differences among the herbal extracts investigated were evaluated using one-way analysis of variance (ANOVA), followed by Tukey’s post hoc multiple comparison test. Statistical significance was set at *p* < 0.05. All statistical analyses were performed using SPSS version 22.0 (IBM, Chicago, USA) [42].

### Pearson correlations and heatmap analysis

Pearson correlation coefficients (*r*) between phenolic constituents, antioxidant capacity, and enzyme inhibitory activities were calculated using Python (version 3.10) with the Pandas and SciPy libraries. Heatmaps were generated using the Seaborn (v0.11.2) visualization package and Matplotlib (v3.4.3). The correlation matrix was used to identify the strength and direction of associations between variables, with results visualized as clustered heatmaps. Color gradients represent the magnitude and polarity of correlations, and annotated cells indicate Pearson *r* values. In addition, statistical significance of correlations was assessed with two-tailed *p*-values, with *p* < 0.05 considered significant. Confidence intervals were also calculated to provide an estimate of variability around *r* values.

## Results and discussion

### Phytochemical analysis and phenolics quantification in Moroccan herbal teas

#### Total phenolic content (TPC) in Moroccan herbal teas

The TPC of the herbal extracts studied demonstrated significant variation among samples (*p* < 0.05), ranging from approximately 246.7 to 883.2 mg GAE/g dw, reflecting diverse phytochemical richness among the species (Table 2), about 3.6-fold between the richest and least rich teas. Herbs such as *Mentha × piperita* (NA) exhibited the highest TPC with 883.2 ± 13.0 mg GAE/g dw, followed closely by *Thymus broussonetti* (TB) at 746.8 ± 22.4 mg GAE/g dw and *Origanum majorana* (OM) at 733.5 ± 20.3 mg GAE/g dw. These values indicate considerable variability in phenolic accumulation among the investigated herbal teas and are generally comparable to, or higher than, those reported previously for several Mediterranean medicinal plants, although direct comparisons should be interpreted cautiously because of differences in extraction procedures, plant origin, and analytical methods [43, 44].

**Table 2:** Total phenolic content (TPC) of the investigated Moroccan herbal teas expressed as mg GAE/g dw, together with the antioxidant activities determined by the DPPH and ABTS radical scavenging assays (IC_50_ µg/mL) and the ferric reducing power assay (FRAP; reducing power, µg/mL). Significant differences among extracts were evaluated by one-way ANOVA followed by Tukey’s post hoc test (*p* < 0.05). Corresponding p values are provided in Suppl. Tables S2–S4. Herbal codes are defined in Table 1

Herbal code	Total phenolic content (mg GAE/g dw) ± SD	IC_50_ (µg/mL)			Reducing power (µg/mL)
		DPPH	ABTS	FRAP	
GG	435.0 ± 4.6	4.0 ± 1.5	225.6 ± 8.3	161.8 ± 1.4	
PG	422.9 ± 3.2	4.0 ± 1.8	238.2 ± 37.4	200.2 ± 5.4	
RO	477.3 ± 9.0	7.3 ± 0.9	254.7 ± 12.1	227.2 ± 0.8	
BD	248.6 ± 4.6	1.2 ± 0.3	193.1 ± 14.3	88.4 ± 2.4	
MC	379.7 ± 13.8	3.7 ± 6.4	217.9 ± 5.0	66.4 ± 10.8	
AA	552.0 ± 5.8	2.6 ± 0.9	321.3 ± 3.8	242.6 ± 7.4	
MP	727.7 ± 7.4	38.2 ± 4.1	436.9 ± 12.7	415.5 ± 27.2	
AL	282.7 ± 5.1	0.8 ± 0.4	189.8 ± 7.7	98.0 ± 2.4	
HS	246.7 ± 4.6	2.4 ± 0.6	114.4 ± 14.9	92.6 ± 3.0	
LC	490.2 ± 2.5	4.8 ± 1.7	263.6 ± 8.8	282.0 ± 1.2	
CO	656.4 ± 9.5	32.6 ± 5.8	390.5 ± 15.6	316.4 ± 1.2	
OM	733.5 ± 20.3	21.4 ± 1.1	461.7 ± 13.3	429.9 ± 4.8	
MV	422.6 ± 6.5	2.6 ± 0.1	251.3 ± 12.2	190.8 ± 26.4	
NA	883.2 ± 13.0	48.1 ± 1.6	645.4 ± 18.9	435.7 ± 17.4	
FV	449.8 ± 4.6	5.4 ± 0.6	231.3 ± 14.5	228.8 ± 2.4	
RI	508.9 ± 0.9	9.6 ± 0.4	308.1 ± 6.7	240.4 ± 8.8	
SO	557.4 ± 19.4	12.0 ± 2.1	325.9 ± 24.5	278.6 ± 5.8	
MR	720.0 ± 19.6	39.8 ± 1.5	451.7 ± 7.8	162.6 ± 0.6	
OC	632.8 ± 11.3	14.7 ± 1.6	341.5 ± 11.1	145.0 ± 4.6	
TB	746.8 ± 22.4	40.1 ± 1.2	518.5 ± 10.0	423.6 ± 9.8	
CA	674.1 ± 7.8	22.6 ± 4.1	427.2 ± 23.4	396.8 ± 6.9	

Similarly, *Mentha pulegium* (MP) and *Mentha rotundifolia* (MR) exhibited high phenolic levels, with 727.7 ± 7.4 and 720.0 ± 19.6 mg GAE/g dw, respectively, confirming the significant phenolic richness of the Lamiaceae family members in this study. Based on the distribution of TPC values, eight herbal teas (NA, TB, OM, MP, MR, CA, CO, and OC) were classified as high-phenolic (> 600 mg GAE/g dw), six teas (RO, SO, LC, FV, PG, and RI) as moderate (400–600 mg GAE/g dw), and seven teas (BD, HS, AL, MC, AA, GG, and MV) as low (< 400 mg GAE/g dw).

To ensure representative coverage of the chemical diversity, one to two teas from each phenolic category were prioritized for subsequent in vitro antioxidant and enzyme inhibition assays. This stratified selection provided a basis for comparing teas with different phenolic contents and exploring whether total phenolic content was associated with the measured bioactivities.

High TPC values were observed for *Citrus aurantium* (CA) (674.1 ± 7.8 mg GAE/g dw), *Calamintha officinalis* (CO) (656.4 ± 9.5 mg GAE/g dw), and *Origanum compactum* (OC) (632.8 ± 11.3 mg GAE/g dw). These values exceed previously reported phenolic contents for related medicinal plants (150–400 mg GAE/g dw), representing approx. 1.5–3.0-fold higher concentrations than those previously reported and highlighting the comparatively high phenolic content of Moroccan herbal teas [43, 45].

Conversely, *Hibiscus sabdariffa* (HS) and *Illicium verum* (BD) displayed relatively lower TPC values (246.7 ± 4.6 and 248.6 ± 4.6 mg GAE/g dw, respectively), indicating considerable interspecies variation in phenolic accumulation among the investigated Moroccan herbal teas rather than a family-specific trend. Geographic and environmental factors, including Moroccan soil composition, climate, altitude, and growing conditions, may also contribute to the elevated phenolic levels observed in several Lamiaceae species [46]. The significant differences among the investigated extracts were confirmed by one-way ANOVA followed by Tukey’s post hoc test (*p* < 0.05), and the corresponding *p*-values are provided in **Suppl. Tables S2**, **S3**, and **S4**. Hence, the wide variation in TPC highlights the substantial phytochemical diversity of the investigated herbal teas, although TPC alone does not necessarily predict their biological activities.

### Quantification of phenolic constituents by HPLC-MS

The HPLC-MS analysis was the primary technique employed to provide a comprehensive chemical fingerprint of the investigated Moroccan herbal teas. This high-resolution approach enabled the precise identification and accurate quantification of 19 targeted phenolic compounds, including flavonoids, phenolic acids, and their derivatives. These compositional data provide a basis for interpreting the differences in biological activities observed among the herbal infusions. The resulting dataset provides a comprehensive phytochemical profile of each herbal infusion and facilitates interpretation of the observed biological activities. To ensure transparency and support the quantitative data presented in Table 3, representative HPLC chromatograms with annotated peak identities are provided in the Supplementary Data (Suppl. Figures S1–S21). In addition, the chromatographic retention times and mass spectral characteristics of the authentic standards used for compound identification are summarized in Suppl. Table S5.

**Table 3:** Identified compounds by HPLC-MS and their quantities expressed as mean in mg/kg **±** SD (*n* = 3) in the investigated Moroccan herbal teas. The herbal codes are listed in Table 1

Compound Herb name	Compounds and concentration detected (mg/kg) ± SD									
	Gallic acid	Catechin	Rutin	Chlorogenic acid	Vanillic acid	Caffeic acid	Quercetin	Syringic acid	*p*-Hydroxy benzoic acid	Rosmarinic acid
GG	0.2 ± 0.0	1.4 ± 0.1	38.9 ± 1.8	256.4 ± 15.0	630.3 ± 28.8	312.6 ± 13.0	0.3 ± 0.0	14.3 ± 0.7	150.4 ± 7.2	3.9 ± 0.2
PG	9745.6 ± 452.2	74.3 ± 3.2	6989.6 ± 317.3	6.07 ± 0.3	1460.2 ± 66.7	629.7 ± 18.0	88.6 ± 4.0	2.0 ± 0.1	9.9 ± 0.4	0.0
RO	1.0 ± 0.0	16.6 ± 0.7	51.5 ± 2.3	2467.0 ± 100.4	10138.5 ± 463.3	12704.1 ± 745.7	0.0	22.5 ± 1.0	1012.2 ± 44.2	10848.7 ± 492.5
BD	72.6 ± 3.5	163.6 ± 6.7	76.2 ± 3.7	1345.2 ± 56.1	470.2 ± 19.1	20020.4 ± 914.9	0.0	30.8 ± 1.3	158.2 ± 6.4	240.0 ± 11.0
MC	1891.6 ± 92.1	0.0	4914.2 ± 190.2	107.5 ± 5.1	377.4 ± 15.7	343.7 ± 9.9	6.9 ± 0.3	51.2 ± 2.1	909.7 ± 36.5	12.9 ± 0.6
AA	308.5 ± 12.0	5.4 ± 0.3	124.1 ± 3.6	6683.7 ± 285.4	64.3 ± 1.8	877.1 ± 51.5	1.7 ± 0.1	0.0	19.6 ± 1.0	672.6 ± 30.7
MP	91.0 ± 2.6	0.0	2122.2 ± 124.6	10353.4 ± 452.4	134.1 ± 7.9	5264.1 ± 240.6	1.3 ± 0.1	7.1 ± 0.3	123.3 ± 5.7	9275.4 ± 423.9
AL	2.0 ± 0.1	0.1 ± 0.0	6.3 ± 0.3	123.5 ± 5.0	744.4 ± 34.2	308.2 ± 14.1	0.0	4.0 ± 0.2	65.8 ± 3.0	28.3 ± 1.4
HS	242.0 ± 11.0	0.3 ± 0.0	861.6 ± 39.4	3081.4 ± 123.5	58.5 ± 1.7	2648.1 ± 131.6	3.9 ± 0.2	59.8 ± 2.7	41.8 ± 1.9	0.0
LC	0.9 ± 0.0		31.1 ± 1.4	45.0 ± 2.2	586.6 ± 34.4	814.7 ± 37.9	0.1 ± 0.0	2.7 ± 0.1	98.2 ± 4.5	11.6 ± 0.6
CO	22.8 ± 1.0	0.2 ± 0.0	7167.8 ± 1.7	19496.8 ± 906.6	235.8 ± 10.8	7972.0 ± 362.0	43.0 ± 2.0	20.2 ± 0.9	67.2 ± 3.1	4177.6 ± 119.9
OM	15.1 ± 0.7	2.1 ± 0.1	157.4 ± 6.6	18.6 ± 0.8	1286.2 ± 58.8	9135.5 ± 417.5	0.0	50.2 ± 2.2	181.0 ± 8.2	13820.8 ± 811.3
MV	1.2 ± 0.1	0.8 ± 0.0	5.1 ± 0.1	42.8 ± 2.0	2353.5 ± 107.6	28704.5 ± 1311.8	0.0	18.0 ± 0.8	5.6 ± 0.3	386.4 ± 17.7
NA	2.6 ± 0.1	15.5 ± 0.7	11.2 ± 0.7	75.6 ± 3.5	10035.6 ± 408.4	501.5 ± 22.9	0.0	0.0	353.6 ± 16.2	93.0 ± 4.2
FV	17.5 ± 0.7	0.0	1245.7 ± 57.0	3932.4 ± 179.7	263.8 ± 11.0	482.2 ± 22.0	0.0	6.2 ± 0.3	22.4 ± 1.0	7.1 ± 0.3
RI	0.3 ± 0.0	0.0	0.9 ± 0.0	138.6 ± 6.3	0.0	14.8 ± 0.6	0.0	0.0	41.5 ± 1.9	0.0
SO	0.0	0.0	12.8 ± 0.5	154.8 ± 6.3	324.0 ± 19.0	4011.2 ± 160.7	0.0	1780.2 ± 88.5	227.0 ± 11.3	10548.3 ± 302.7
MR	139.4 ± 7.0	1.9 ± 0.1	606.3 ± 29.0	1731.1 ± 72.2	224.7 ± 10.3	8616.2 ± 350.7	0.4 ± 0.0	30.9 ± 0.9	426.9 ± 12.3	10769.0 ± 535.2
OC	45.9 ± 2.1	0.0	1938.2 ± 88.6	218.4 ± 6.3	477.1 ± 21.8	1121.5 ± 44.9	0.0	0.30 ± 0.0	12.3 ± 0.7	0.0
TB	119.7 ± 5.4	1.0 ± 0.0	663.130.3	2324.9 ± 136.5	388.6 ± 17.8	12360.2 ± 614.3	1.9 ± 0.1	118.3 ± 5.4	242.7 ± 11.1	12481.4 ± 732.7
CA	44.1 ± 2.0	0.1 ± 0.0	107.1 ± 4.9	649.4 ± 29.7	3901.1 ± 158.8	10097.6 ± 469.5	0.3 ± 0.0	118.7 ± 5.4	313.3 ± 14.3	13415.7 ± 613.1

Compound Herb name									
	Sinapic acid	Epicatechin	Coumaric acid	Tannic acid	Salicylic acid	Resveratrol	Pyrocatechol	Pyrogallol	Ferulic acid
GG	5.7 ± 0.3	0.1 ± 0.0	39.2 ± 1.1	0.0	4.6 ± 0.1	2.6 ± 0.1	8.9 ± 0.3	12.9 ± 0.6	4.4 ± 0.3
PG	30.2 ± 1.5	84.2 ± 4.2	15.5 ± 0.9	28148.1 ± 807.8	29.6 ± 1.7	19.0 ± 0.9	0.0	176.9 ± 8.2	3.6 ± 0.1
RO	6.7 ± 0.2	89.4 ± 2.6	131.5 ± 6.0	0.0	13.6 ± 0.6	1.4 ± 0.0	0.0	0.8 ± 0.0	188.5 ± 11.1
BD	88.2 ± 4.4	320.9 ± 18.8	28.7 ± 1.4	0.0	5.8 ± 0.3	0.0	0.0	0.0	8.3 ± 0.4
MC	8.3 ± 0.2	6.1 ± 0.3	109.7 ± 3.1	0.0	5.5 ± 0.3	3.8 ± 0.2	92.4 ± 4.2	3.4 ± 0.2	93.6 ± 4.7
AA	197.8 ± 9.0	10.6 ± 0.5	6.4 ± 0.3	0.0	4.0 ± 0.1	0.0	0.0	2.4 ± 0.1	2.6 ± 0.1
MP	3.0 ± 0.1	0.0	59.4 ± 1.7	0.0	9.2 ± 0.3	0.8 ± 0.0	0.0	0.0	10.0 ± 0.5
AL	3.8 ± 0.1	0.0	16.1 ± 0.9	0.0	2.6 ± 0.2	0.0	0.0	0.7 ± 0.0	6.5 ± 0.2
HS	6.0 ± 0.3	0.0	16.2 ± 0.7	0.0	16.7 ± 0.8	0.3 ± 0.0	0.0	0.0	11.4 ± 0.7
LC	0.0	0.0	14.1 ± 0.7	0.0	5.9 ± 0.3	0.8 ± 0.0	0.0	0.0	166.6 ± 7.6
CO	2.5 ± 0.1	0.0	90.8 ± 2.6	0.0	19.6 ± 0.6	1.7 ± 0.1	0.0	0.2 ± 0.0	12.6 ± 0.6
OM	0.9 ± 0.0	4.0 ± 0.1	15.7 ± 0.8	228.7 ± 6.6	8.1 ± 0.4	0.0	0.0	0.1 ± 0.0	51.5 ± 2.6
MV	2.1 ± 0.1	0.0	72.6 ± 2.1	0.0	7.1 ± 0.2	0.0	0.0	2.6 ± 0.1	162.1 ± 4.6
NA	14.1 ± 0.4	14.5 ± 0.7	12.3 ± 0.6	179.7 ± 8.2	0.0	0.0	0.0	0.0	6.8 ± 0.2
FV	0.0	0.0	39.1 ± 1.9	184.6 ± 9.2	2.0 ± 0.1	3.7 ± 0.2	0.0	1.8 ± 0.1	7.2 ± 0.4
RI	0.5 ± 0.0	0.0	4.7 ± 0.1	0.0	15.9 ± 0.5	0.0	0.0	9.7 ± 0.5	1.3 ± 3.7
SO	0.0	1.6 ± 0.1	20.6 ± 0.9	207.4 ± 12.2	8.3 ± 0.5	0.0	0.8 ± 0.0	0.6 ± 0.0	42.9 ± 3.7
MR	109.2 ± 5.4	5.9 ± 0.3	138.6 ± 4.0	128.4 ± 5.9	19.1 ± 0.9	0.1 ± 0.0	0.0	6.9 ± 0.3	143.7 ± 7.2
OC	3.4 ± 0.1	0.0	6.6 ± 0.4	5.5 ± 0.3	0.3 ± 0.0	6.1 ± 0.3	0.0	7.1 ± 0.2	270.7 ± 13.5
TB	5.5 ± 0.3	0.0	9.5 ± 0.4	186.8 ± 9.3	6.8 ± 0.2	1.5 ± 0.0	4.1 ± 0.1	1.3 ± 0.1	25.4 ± 0.1
CA	7.0 ± 0.3	0.0	15.4 ± 0.8	1117.6 ± 32.1	2.1 ± 0.1	0.6 ± 0.0	5.0 ± 0.2	1.3 ± 0.1	9.4 ± 6.1

### Phenolic acid composition

The quantitative HPLC-MS analysis revealed substantial interspecific variation in phenolic acid composition among the 21 investigated herbal infusions. Rosmarinic acid was one of the predominant phenolic acids, particularly among several Lamiaceae species. The highest concentrations were detected in *Origanum majorana* (OM) (13,820.8 ± 811.3 mg/kg), followed by *Citrus aurantium* (CA) (13,415.7 ± 613.1 mg/kg), *Thymus broussonetti* (TB) (12,481.4 ± 732.7 mg/kg), *Rosmarinus officinalis* (RO) (10,848.7 ± 492.5 mg/kg), *Mentha rotundifolia* (MR) (10,769.0 ± 535.2 mg/kg), *Salvia officinalis* (SO) (10,548.3 ± 302.7 mg/kg), and *Mentha pulegium* (MP) (9,275.4 ± 423.9 mg/kg). In contrast, rosmarinic acid was absent or detected only at trace levels in several non-Lamiaceae species, highlighting pronounced species-dependent differences in its accumulation. The marked differences among species suggest that the contribution of individual phenolic acids to the biological activities of the infusions may be strongly species-dependent.

Caffeic acid also showed remarkable variation among the herbal teas investigated. The highest concentration was recorded in *Mentha viridis* (MV) (28,704.5 ± 1311.8 mg/kg), followed by *Illicium verum* (BD) (20,020.4 ± 914.9 mg/kg), *Rosmarinus officinalis* (RO) (12,704.1 ± 745.7 mg/kg), *Thymus broussonetti* (TB) (12,360.2 ± 614.3 mg/kg), *Citrus aurantium* (CA) (10,097.6 ± 469.5 mg/kg), and *Origanum majorana* (OM) (9,135.5 ± 417.5 mg/kg). These findings demonstrate considerable variation in hydroxycinnamic acid accumulation among Moroccan herbal teas and are consistent with previous reports describing caffeic acid as an abundant constituent of several medicinal plants belonging to the Lamiaceae family [47]. However, the presence of high concentrations of a particular phenolic compound does not by itself establish its contribution to the observed biological activities.

### Distribution of other phenolic acids

Chlorogenic acid exhibited one of the widest concentration ranges among the investigated species. The highest levels were detected in *Calamintha officinalis* (CO) (19,496.8 ± 906.6 mg/kg), followed by *Mentha pulegium* (MP) (10,353.4 ± 452.4 mg/kg), *Artemisia absinthium* (AA) (6,683.7 ± 285.4 mg/kg), *Foeniculum vulgare* (FV) (3,932.4 ± 179.7 mg/kg), and *Hibiscus sabdariffa* (HS) (3,081.4 ± 123.5 mg/kg), whereas *Pelargonium graveolens* (PG) contained only trace amounts (6.07 ± 0.3 mg/kg).

Vanillic acid was particularly abundant in *Rosmarinus officinalis* (RO) (10,138.5 ± 463.3 mg/kg) and *Mentha × piperita* (NA) (10,035.6 ± 408.4 mg/kg), followed by *Citrus aurantium* (CA) (3,901.1 ± 158.8 mg/kg) and *Mentha viridis* (MV) (2,353.5 ± 107.6 mg/kg). Overall, the heterogeneous distribution of individual phenolic acids reflects species-specific metabolic characteristics that may also be influenced by environmental conditions, geographical origin, and genetic background. Previous studies have shown that environmental factors, including soil composition, altitude, and climatic conditions, significantly influence the biosynthesis and accumulation of secondary metabolites in Moroccan medicinal plants [46].

### Flavonoid composition

Among the quantified flavonoids, rutin was the predominant compound in *Calamintha officinalis* (CO) (7,167.8 ± 1.7 mg/kg), *Pelargonium graveolens* (PG) (6,989.6 ± 317.3 mg/kg), *Matricaria chamomilla* (MC) (4,914.2 ± 190.2 mg/kg), *Mentha pulegium* (MP) (2,122.2 ± 124.6 mg/kg), and *Origanum compactum* (OC) (1,938.2 ± 88.6 mg/kg). Epicatechin occurred at comparatively lower concentrations, with the highest levels detected in *Illicium verum* (BD) (320.9 ± 18.8 mg/kg), followed by *Rosmarinus officinalis* (RO) (89.4 ± 2.6 mg/kg) and *Pelargonium graveolens* (PG) (84.2 ± 4.2 mg/kg). Resveratrol was detected only at low concentrations, with *Pelargonium graveolens* (PG) exhibiting the highest level (19.0 ± 0.9 mg/kg). Thus, these findings demonstrate substantial variability in flavonoid composition among Moroccan herbal teas, which may contribute to the differences observed in antioxidant and enzyme inhibitory activities, although such relationships cannot be attributed to individual compounds based solely on their concentrations.

### Antioxidant activity (DPPH, ABTS, and FRAP assays)

The antioxidant potential of the studied herbal teas was evaluated using three complementary assays, i.e., DPPH, ABTS, and FRAP, to ensure reliable assessment of both radical scavenging and reducing capacities. The DPPH and ABTS assays assess the ability of extracts to neutralize free radicals by bleaching their respective radical species, whereas FRAP measures the electron-donating power through ferric ion reduction. Thus, these assays evaluate two major antioxidant mechanisms, namely hydrogen atom transfer (HAT) and single electron transfer (SET), reflecting the redox behavior of plant phenolics under in vitro conditions without directly modeling biologically relevant reactive oxygen species such as superoxide or hydroxyl radicals.

The results presented in Table 2 demonstrate marked variability in antioxidant capacity among the tested teas, highlighting the influence of both phenolic content and composition. Some species with high TPC (TPC > 700 mg GAE/g dw), such as *Mentha × piperita* (NA, 883.2 ± 13.0), *Origanum majorana* (OM, 733.5 ± 20.3), *Thymus broussonetii* (TB, 746.8 ± 22.4), and *Mentha pulegium* (MP, 727.7 ± 7.4), did not display proportionally strong antioxidant activities.

For instance, NA exhibited relatively high IC_50_ values in the DPPH and ABTS assays (48.1 ± 1.6 and 645.4 ± 18.9 µg/mL, respectively), indicating comparatively weaker radical scavenging activity despite its high phenolic content. Similarly, MP (38.2 ± 4.1 and 436.9 ± 12.7 µg/mL for DPPH and ABTS, respectively) and TB (40.1 ± 1.2 and 518.5 ± 10.0 µg/mL, respectively) also exhibited relatively high IC₅₀ values, suggesting lower antioxidant efficiency relative to their total phenolic contents.

In contrast, species with relatively low TPC values, including *Illicium verum* (BD, 248.6 ± 4.6 mg GAE/g dw), *Ammodaucus leucotrichus* (AL, 282.7 ± 5.1 mg GAE/g dw), and *Hibiscus sabdariffa* (HS, 246.7 ± 4.6 mg GAE/g dw), displayed comparatively low IC_50_ values in both DPPH (BD = 1.2 ± 0.3, AL = 0.8 ± 0.4, and HS = 2.4 ± 0.6 µg/mL) and ABTS assays (BD = 193.1 ± 14.3, AL = 189.8 ± 7.7, and HS = 114.4 ± 14.9 µg/mL), indicating stronger antioxidant activity than several phenolic-rich extracts.

These findings indicate that TPC alone is not a reliable predictor of antioxidant performance in the investigated teas. The stronger activity observed in some samples with relatively lower TPC suggests that the identity, relative abundance, and possible interactions of individual constituents may be more important determinants of antioxidant capacity than total phenolic concentration alone [48]. Therefore, while TPC provides a useful estimate of overall phenolic richness, the present findings suggest that antioxidant capacity is influenced by qualitative composition and possible interactions among phenolics and other constituents.

The herbal infusions from the Lamiaceae family, particularly those represented by NA, TB, OM, and MP, showed considerable variation in antioxidant activity across the DPPH, ABTS, and FRAP assays, indicating that high TPC does not necessarily translate into stronger antioxidant activity. Instead, the antioxidant performance appears to depend on the composition and relative abundance of individual phenolic constituents rather than their total concentration alone. Phenolics have demonstrated considerable potential in diabetes control, especially due to their antioxidant abilities. They neutralize free radicals and mitigate oxidative stress, a primary factor in diabetes pathogenesis [49, 50]. Although the current *in vitro assays* do not directly measure superoxide or cellular-level antioxidant responses, they provide a valuable biochemical indication of redox potential relevant to diabetes-related oxidative mechanisms. Phenolic compounds have also been reported to modulate oxidative stress-related pathways, including nuclear factor-κB and mitogen-activated protein kinases, which are associated with inflammation and insulin resistance [49]. However, these mechanisms were not directly investigated in the present study.

### Enzymatic inhibition activities

**Fig. 1:**
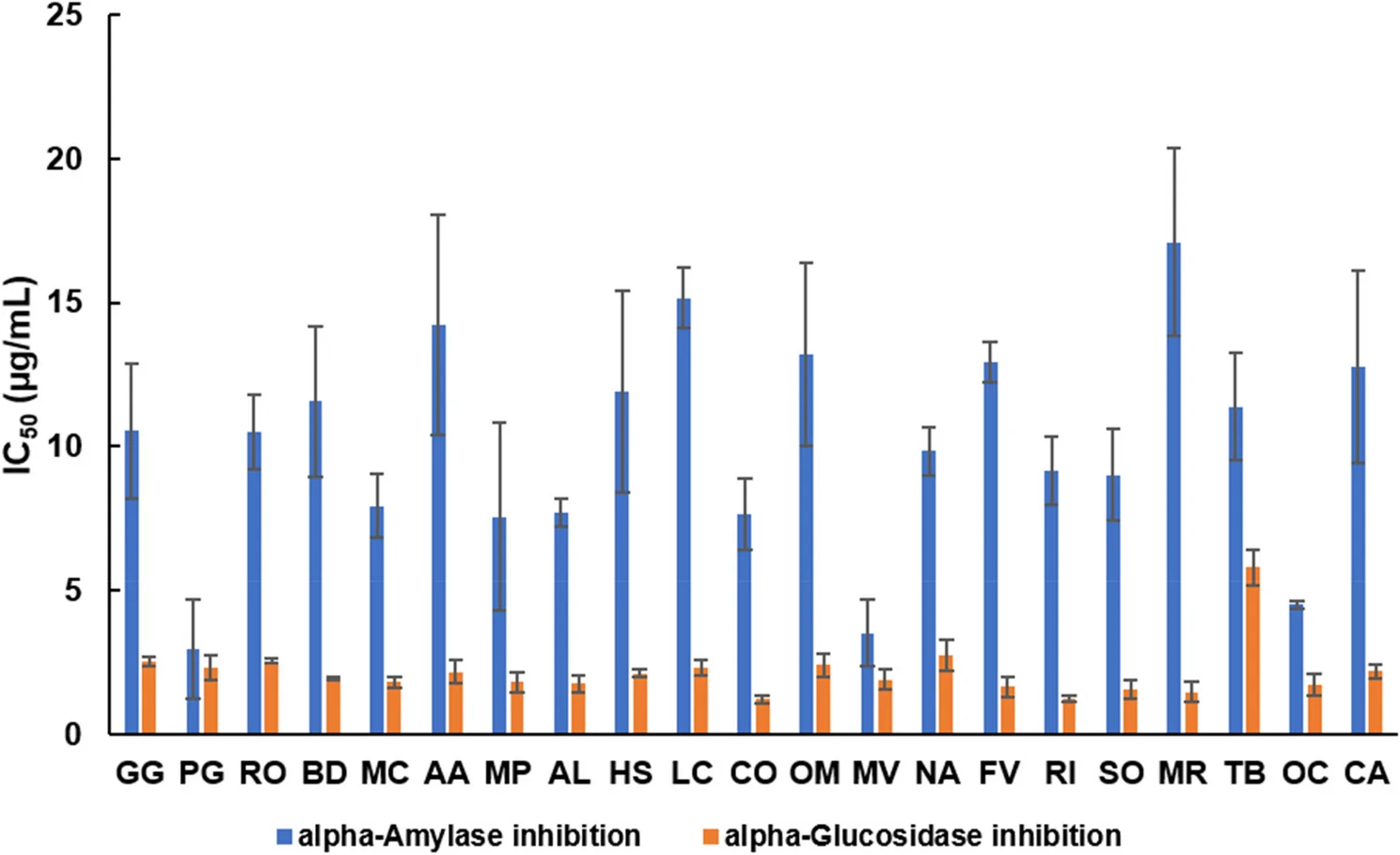
Enzymatic inhibition activity expressed as IC_50_ (µg/mL) of the investigated herbal teas against *α*-amylase and *α*-glucosidase enzymes

Effective antidiabetic agents should possess strong inhibitory activity against key enzymes involved in postprandial glucose elevation. In this study, the herbal extracts were evaluated in vitro for their ability to inhibit *α*-amylase and *α*-glucosidase, the two primary enzymes responsible for carbohydrate digestion. The results obtained were demonstrated in Table 4; Fig. 1. Significant differences among extracts were evaluated using one-way ANOVA followed by the appropriate post hoc test at *p* < 0.05. The *p*-values are provided in Suppl. Tables S6 and **S7**.

**Table 4:** A summarized overview of enzymes inhibition activity showing the IC_50_ (µg/mL) ± SD (*n* = 3) of amylase and glucosidase of the investigated Moroccan herbal teas. The significant difference between extracts was tested using one way ANOVA followed by post HOC test at *p* < 0.05. The *p* values are provided in Suppl. Table S6 and S7. The herbal codes are listed in Table 1

Herbal codes	α-Amylase inhibition	α-Glucosidase inhibition
	IC_50_ (µg/mL) ± SD*	
GG	10.5 ± 2.4	2.5 ± 0.2
PG	2.98 ± 1.7	2.3 ± 0.4
RO	10.5 ± 1.3	2.5 ± 0.1
BD	11.6 ± 2.6	1.9 ± 0.1
MC	7.9 ± 1.1	1.8 ± 0.2
AA	14.2 ± 3.8	2.2 ± 0.4
MP	7.6 ± 3.3	1.8 ± 0.3
AL	7.7 ± 0.5	1.8 ± 0.3
HS	11.9 ± 3.5	2.1 ± 0.1
LC	15.2 ± 1.1	2.3 ± 0.3
CO	7.7 ± 1.2	1.2 ± 0.1
OM	13.2 ± 3.2	2.4 ± 0.4
MV	3.5 ± 1.2	1.9 ± 0.4
NA	9.8 ± 0.8	2.8 ± 0.5
FV	12.9 ± 0.7	1.6 ± 0.3
RI	9.2 ± 1.2	1.2 ± 0.1
SO	9.0 ± 1.6	1.6 ± 0.3
MR	17.1 ± 3.3	1.5 ± 0.4
TB	11.4 ± 1.9	1.7 ± 0.4
OC	4.5 ± 0.1	5.8 ± 0.6
CA	12.8 ± 3.4	2.2 ± 0.3

*IC_50_ values were calculated from concentration-response curves generated using two-fold serial dilutions. Preliminary screening experiments were first conducted to identify the concentration range producing approximately 50% enzyme inhibition, after which additional lower concentrations were included when necessary to ensure accurate IC₅₀ determination

GG (*Glycyrrhiza glabra*), PG (*Pelargonium graveolens*), RO (*Rosmarinus officinalis*), BD (*Illicium verum*), MC (*Matricaria chamomilla*), AA (*Artemisia absinthium*), MP (*Mentha pulegium*), AL (*Ammodaucus leucotrichus*), HS (*Hibiscus sabdariffa*), LC (*Lippia citriodora*), CO (*Calamintha officinalis*), OM (*Origanum majorana*), MV (*Mentha viridis*), NA (*Mentha x piperita*), FV (*Foeniculum vulgare*), RI (*Myrtus communis*), SO (*Salvia officinalis*), MR (*Mentha rotundifolia*), and OC (*Origanum compactum*).

### Alpha-amylase inhibition activity

The results shown in Table 4; Fig. 1 demonstrated that *Pelargonium graveolens* (PG) and *Mentha viridis* (MV) exhibited the strongest *α*-amylase inhibitory activities, with IC_50_ values of 2.98 ± 1.7 and 3.5 ± 1.2 µg/mL, respectively. HPLC analysis indicated that PG is rich in gallic acid, rutin, and tannic acid, while MV contains vanillic acid and caffeic acid. *α*-Amylase catalyzes the hydrolysis of *α*-1,4-glycosidic linkages in starch into glucose, and its inhibition is a key strategy to reduce postprandial glucose surges [51–53].

PG and MV exhibited the strongest *α*-amylase inhibition among the investigated teas. Their high activity may be associated with their complex phenolic composition rather than the abundance of a single metabolite. However, the present data do not establish whether these compounds act additively or synergistically. Previous studies have likewise demonstrated that crude plant extracts frequently exhibit stronger enzyme inhibition than isolated constituents because of complementary mechanisms of action [54–56].

### Alpha-glucosidase inhibition activity

The investigated herbal teas revealed relatively similar inhibition profiles against *α*-glucosidase as demonstrated in Table 4; Fig. 1, with *Calamintha officinalis* (CO) and *Matricaria chamomilla* (MC) exhibited the most potent activity with IC_50_ values of 1.103 ± 0.1 and 1.108 ± 0.1 µg/mL, respectively. These activities may be associated with the presence of caffeic acid, chlorogenic acid, rutin, vanillic acid, and rosmarinic acid. *α*-Glucosidase inhibitors delay carbohydrate hydrolysis, reducing postprandial glucose absorption and glycated hemoglobin (HbA1c) levels [57–59].

The strong *α*-glucosidase inhibitory activity observed for CO and MC is consistent with their complex phenolic composition revealed by HPLC-MS analysis. Several of the quantified phenolic acids and flavonoids identified in these extracts, including caffeic acid, chlorogenic acid, rutin, vanillic acid and rosmarinic acid, have previously been reported as *α*-glucosidase inhibitors. Therefore, the observed inhibitory activity may reflect the contribution of multiple phenolic constituents rather than a single dominant metabolite, although their individual or combined contributions cannot be established from the present data alone [60–64].

Therefore, the integrated phytochemical and biological analyses demonstrated considerable interspecific variability among the investigated Moroccan herbal teas. While TPC showed strong correlations with antioxidant activity, its relationship with enzyme inhibitory activity was less consistent, highlighting the importance of individual phenolic composition in determining biological activity. The HPLC-MS profiling further revealed distinct chemical fingerprints for each species, supporting the value of combining targeted metabolite quantification with functional bioassays for the scientific evaluation of traditional herbal teas.

### Correlations between phenolic content and antioxidant activity

Pearson correlation was selected over multivariate methods such as principal component analysis (PCA) since it provides a direct measure of linear dependence between phenolic composition and biological responses. Pearson correlation coefficients (*r*) were calculated to quantify the degree of association between variables, while PCA was deemed less suitable because the primary objective was not dimensional reduction but the assessment of specific pairwise relationships among phenolic constituents and biological parameters [65, 66].

**Fig. 2:**
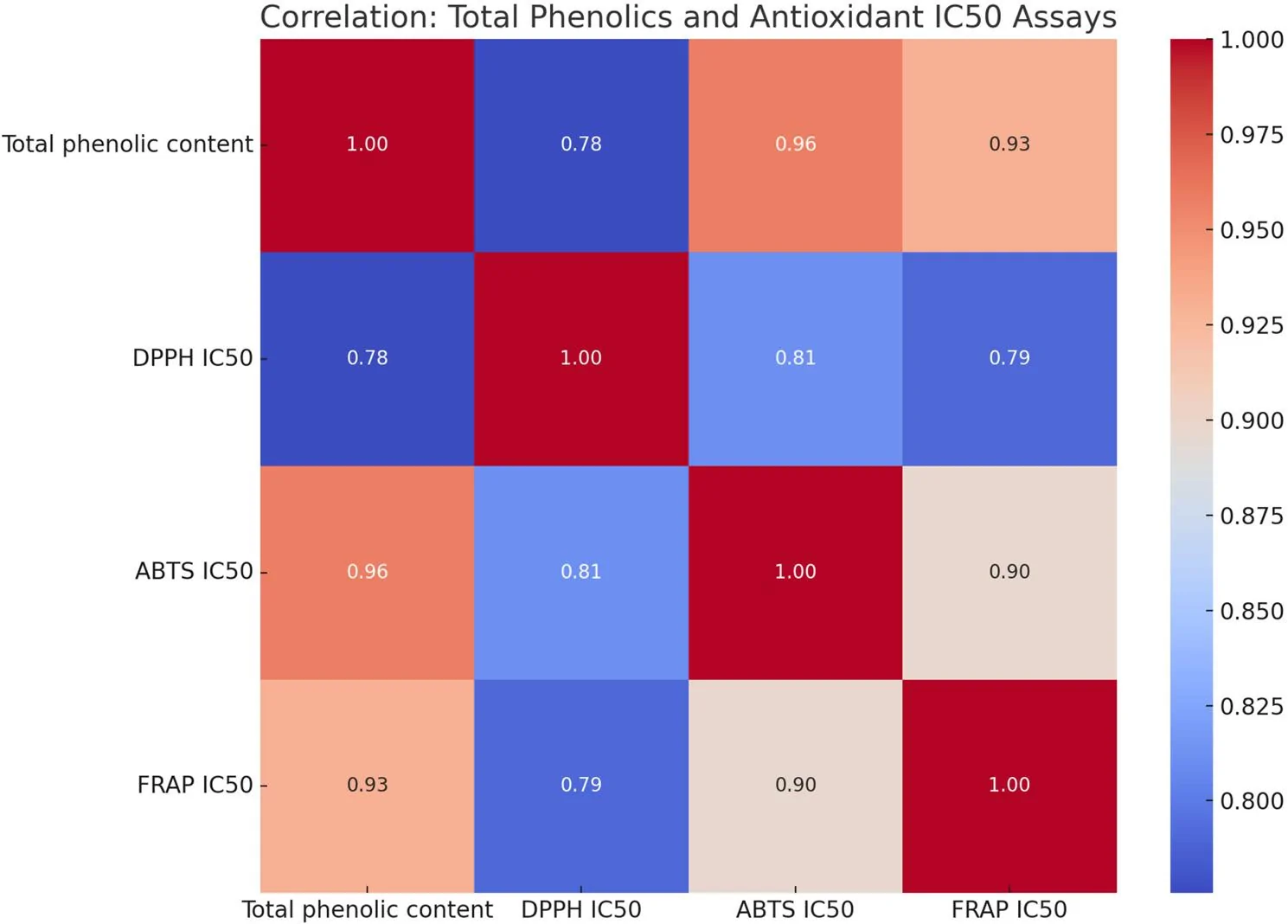
Heatmap showing the Pearson correlation coefficients between total phenolic content (TPC) and antioxidant activity assays (DPPH and ABTS IC_50_ values and FRAP reducing power values). The heatmap illustrates strong positive correlations between TPC and antioxidant activity, with the highest correlation observed between TPC and ABTS (*r* = 0.96). Blue shades represent negative correlations, whereas red shades represent positive correlations, with correlation coefficients displayed inside each cell

The TPC of Moroccan herbal teas exhibited strong positive correlations with their antioxidant capacities measured by three independent assays. Pearson correlation analysis revealed that TPC was highly correlated with ABTS (*r* = 0.96) and FRAP reducing power (*r* = 0.93) values, and to a slightly lesser extent with DPPH (*r* = 0.78), as illustrated in Fig. 2. These findings support an important association between total phenolic content and the antioxidant capacity of these infusions.

Herbs such as *Mentha × piperita* (NA), *Thymus broussonetii* (TB), and *Mentha rotundifolia* (MR) exhibited both high phenolic concentrations and strong antioxidant activity, indicating the contribution of active constituents such as caffeic, chlorogenic, and rosmarinic acids, which are well recognized for their redox potential and hydrogen-donating ability.

Hence, these results indicate that several phenolic-rich Moroccan herbal teas exhibit strong in vitro antioxidant activity, although the clinical relevance of these findings requires further investigation. Furthermore, the close alignment between phenolic levels and antioxidant activity suggests the potential of total phenolic content as a reliable proxy for screening antioxidant efficacy in traditional herbal formulations.

### Correlations between phenolic constituents and enzyme inhibitory activity

Enzyme inhibition, particularly of *α*-amylase and *α*-glucosidase, is critical for management of T2DM. The current study assessed the correlation between individual phenolic compound concentrations and enzyme inhibitory activity, expressed as IC_50_ values, revealing strong and biologically meaningful patterns.

**Fig. 3:**
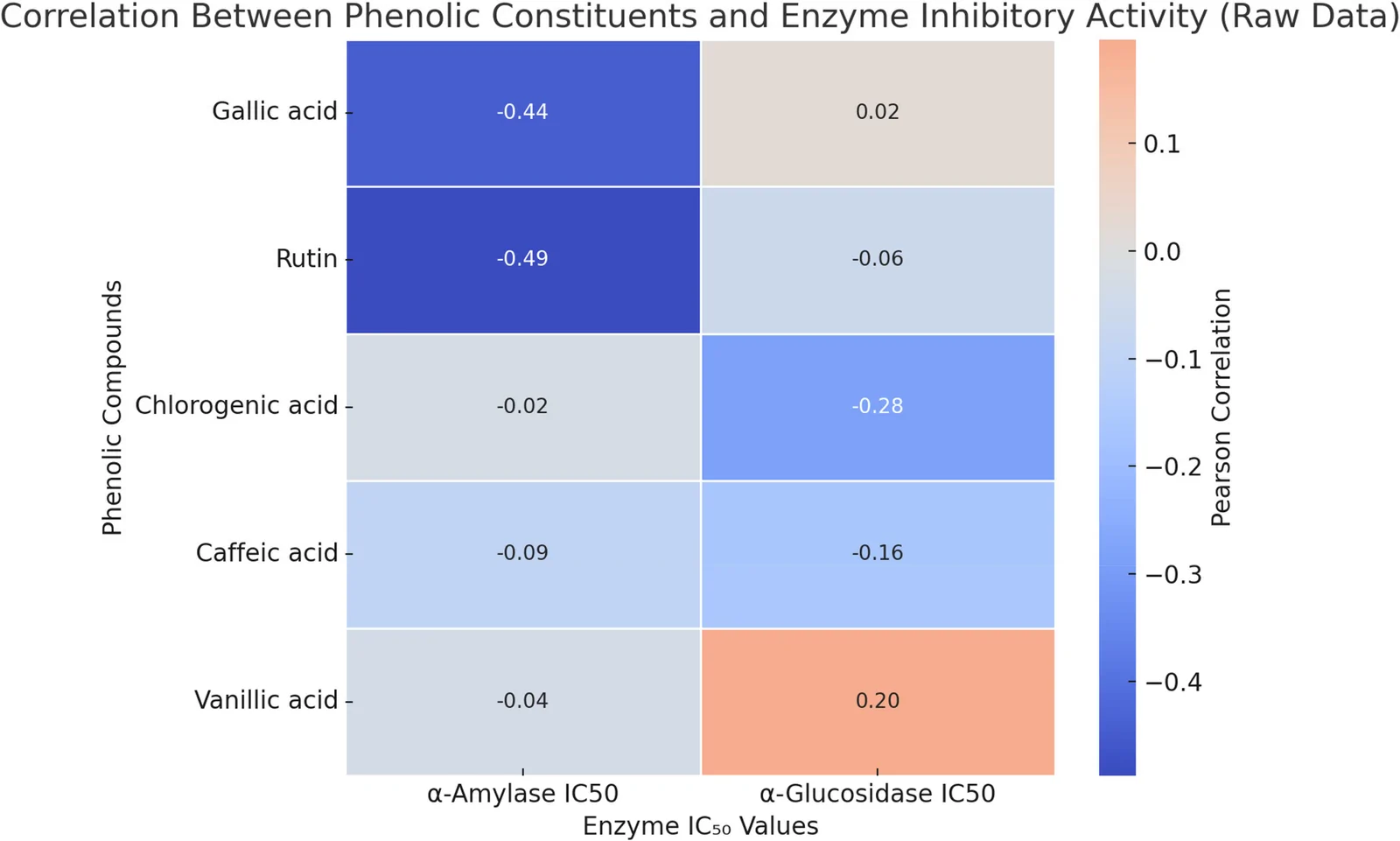
Heatmap depicting Pearson correlation coefficients between major phenolic compounds (mg/kg) and enzyme inhibitory activity (IC_50_ values in µg/mL) against *α*-amylase and *α*-glucosidase in Moroccan herbal teas. Negative correlations (blue) indicate that higher compound concentrations are associated with stronger enzyme inhibition (lower IC_50_), while positive correlations (red) suggest weaker inhibition or potential antagonistic effects. Gallic acid and rutin show moderate inverse correlations with α-amylase inhibition, highlighting their potential contributions to antidiabetic activity

Pearson correlation analysis using raw data revealed moderate negative correlations between gallic acid (*r* = − 0.44, *p* = 0.032) and rutin (*r* = − 0.49, *p* = 0.021) with *α*-amylase IC_50_ values, suggesting that these phenolic compounds may contribute to the observed *α*-amylase inhibitory activity, Fig. 3. In contrast, their associations with *α*-glucosidase inhibition were minimal (*r* = + 0.02 and − 0.06, respectively), indicating potential enzyme specificity or the influence of synergistic effects. Chlorogenic acid showed a weak negative correlation with *α*-amylase IC_50_ (*r* = − 0.02) but a more noteworthy inverse association with *α*-glucosidase IC_50_ (*r* = − 0.28), implying a more relevant role in glucosidase targeting. Caffeic acid followed a similar trend (*r* = − 0.09 with *α*-amylase, *r* = − 0.16 with *α*-glucosidase). Interestingly, vanillic acid demonstrated only negligible correlation with α-amylase (*r* = − 0.04), but a modest positive correlation with *α*-glucosidase IC_50_ (*r* = + 0.20), suggesting a possible antagonistic or non-specific role, Fig. 3. These patterns emphasize that individual compound effects must be considered within the broader phytochemical matrix, where interactions may enhance or suppress bioactivity [67, 68].

The negative correlations indicate that higher concentrations of rutin and gallic acid were associated with lower *α*-amylase IC_50_ values and, consequently, stronger inhibition. These observations are consistent with previous studies reporting the inhibitory effects of these phenolic compounds against carbohydrate-digesting enzymes [69, 70].

### Integrative analysis: Phenolic content, constituents, and enzyme inhibition in diabetes management

**Fig. 4:**
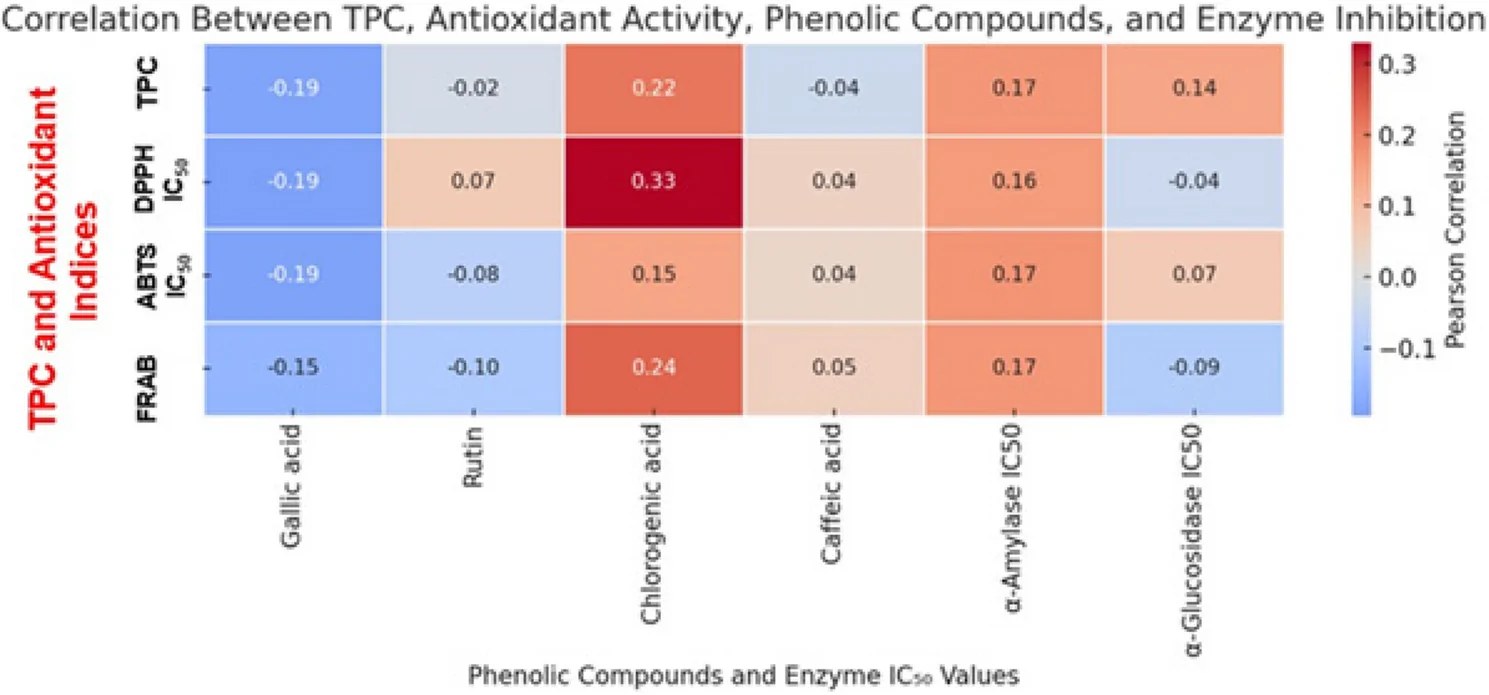
Pearson correlation heatmap between total phenolic content (TPC), antioxidant activities (DPPH and ABTS IC₅₀ values, FRAP reducing power), phenolic compounds (gallic acid, rutin, chlorogenic acid, caffeic acid), and enzyme inhibitory activities (α-amylase and *α*-glucosidase IC_50_ values) in Moroccan herbal teas. Negative correlations (blue) indicate a potential enhancement of bioactivity with increased compound concentration (e.g., lower IC_50_ values for enzyme inhibition), whereas positive correlations (red) may suggest weaker or indirect relationships

Integrating TPC, individual phenolic compound profiles, and enzyme inhibition data provided a more comprehensive understanding of the relationships between phytochemical composition and enzyme inhibition. While TPC demonstrated strong correlations with antioxidant activity (e.g., *r* = 0.96 with ABTS and *r* = 0.93 with FRAP reducing power, both *p* < 0.01), its relationship with digestive enzyme inhibition was comparatively weak. Instead, specific compounds, particularly rutin and gallic acid, demonstrated moderate negative correlations with *α*-amylase IC_50_ (*r* = − 0.49, *p* = 0.021 and *r* = − 0.44, *p* = 0.032, respectively), indicating that higher concentrations of these phenolics are generally associated with enhanced enzyme inhibition, Fig. 4. This pattern was evident in bioactive-rich species such as *Calamintha officinalis* (CO), *Pelargonium graveolens* (PG), and *Mentha viridis* (MV), which combined elevated phenolic levels with low IC_50_ values for *α*-amylase and *α*-glucosidase.

These findings align with previous reports suggesting that flavonoids and hydroxybenzoic acids act as multitarget antidiabetic agents by directly inhibiting carbohydrate-digestive enzymes and modulating oxidative stress pathways [71, 72], although the present study did not directly investigate their molecular targets or intracellular mechanisms. Interestingly, chlorogenic acid and caffeic acid showed weaker or inconsistent correlations with enzyme inhibition, indicating the complexity of phytochemical interactions and the potential for synergistic or antagonistic effects within the extract matrix.

Hence, these findings provide experimental support for the traditional use of these Moroccan herbal teas and provide a basis for further investigation of polyphenol-enriched formulations.

### Influence of environmental and agronomic factors on bioactive variability

The observed variability in phenolic content and bioactivity among the studied Moroccan herbal teas may be influenced by multiple environmental and agronomic factors. Geographic origin is a key determinant, as soil composition, altitude, climate, and sunlight exposure can significantly affect secondary metabolite biosynthesis, including polyphenols and flavonoids [49, 50]. Seasonal fluctuations and the timing of harvest further modulate the accumulation of phenolic compounds, as plant metabolism responds dynamically to temperature, photoperiod, and water availability [73, 74]. Additionally, the phenological stage of the plant at the time of collection can alter the relative abundance of specific bioactive compounds, potentially impacting antioxidant capacity and enzyme inhibition activity [75, 76]. Although the current study did not experimentally control these variables, acknowledging their influence provides a plausible explanation for the inter-sample variability observed and highlights the importance of standardizing plant material collection for reproducible pharmacological evaluation. Future studies may benefit from systematically investigating these factors to better correlate environmental conditions with bioactive potential.

### Conclusion and future outlook

This study provides scientific support for the traditional use of Moroccan herbal teas in the context of T2DM management. Through integrated phytochemical profiling, antioxidant assessments, and enzyme inhibition assays, the investigated herbal infusions demonstrated variable antioxidant and inhibitory activities against *α*-amylase and *α*-glucosidase, which were generally associated with their phenolic composition. Among the twenty-one teas examined, *Pelargonium graveolens*,* Mentha viridis*,* Calamintha officinalis*, and *Matricaria chamomilla* consistently emerged as the most bioactive species, combining low IC_50_ values against digestive enzymes with high concentrations of key phenolics such as rutin, gallic, chlorogenic, and caffeic acids. These findings expand upon previous studies by providing comparative phytochemical and biological data for Moroccan herbal teas prepared as infusions, thereby better reflecting traditional consumption practices. Moderate negative correlations between rutin and gallic acid concentrations and α-amylase IC_50_ values further support their potential contribution to enzyme inhibition, while strong positive correlations between antioxidant capacity and total phenolic content highlight the multifunctional role of these phytochemicals.

The novelty of this work lies in its comprehensive integration of phytochemical characterization, antioxidant evaluation, and enzyme inhibitory activities across a diverse panel of Moroccan herbal teas using standardized infusion-based extracts and correlation analyses. This integrated approach contributes to bridging traditional ethnobotanical knowledge with experimental biochemical evidence.

Nevertheless, several limitations should be acknowledged. The present study is limited to in vitro biochemical assays, which cannot directly predict in vivo efficacy or clinical relevance. In addition, IC_50_ values were estimated using linear regression within the linear portion of the concentration-response curve. Future studies should employ non-linear concentration-response modeling with confidence intervals and goodness-of-fit statistics for more robust IC_50_ estimation. Furthermore, the concentrations producing enzyme inhibition in vitro may not necessarily be achievable following dietary consumption of herbal infusions. Variability associated with geographic origin, environmental conditions, seasonal harvesting, and plant developmental stage may also influence phytochemical composition and biological activity. Moreover, inflammatory biomarkers, including TNF-α, IL-1β, IL-6, PGE2, and NF-κB-related markers, were not measured in the present study, limiting the direct assessment of the underlying anti-inflammatory mechanisms.

Future research should prioritize in vivo validation, pharmacokinetic and bioavailability studies, and structure-activity relationship investigations to confirm efficacy and safety under physiological conditions. Measurement of inflammatory mediators and NF-κB-related markers should also be considered in future studies to strengthen the mechanistic interpretation of the observed biological activities. Further investigation of synergistic interactions among phenolic constituents, as well as their interactions with the gut microbiota, will improve understanding of the mechanisms underlying the observed bioactivities. In addition, integrating molecular docking and molecular dynamics simulations with experimental validation may provide further mechanistic insights into ligand-enzyme interactions and help identify the phenolic constituents primarily responsible for *α*-amylase and *α*-glucosidase inhibition. Finally, standardization of extraction procedures and development of consumer-ready formulations will be essential to facilitate the translation of these findings into functional foods and nutraceutical applications.

Therefore, this study provides a comprehensive phytochemical and biological evaluation of traditionally consumed Moroccan herbal teas and highlights several species as promising sources of bioactive phenolics with antioxidant and digestive enzyme inhibitory properties. These findings support the potential of polyphenol-rich herbal infusions as complementary dietary strategies for T2DM management, but do not establish their therapeutic efficacy. Further mechanistic, pharmacological, and clinical investigations are required to confirm their efficacy, safety, and clinical relevance.

## Supplementary Information

Supplementary Material 1.

## Data Availability

All data generated or analysed during this study are included in this published article and its supplementary information files.
