# Supplementary material for: Ethnopharmacological insights into type 2 diabetes management by traditional Moroccan herbal teas: HPLC-MS characterization of phenolic compounds and their related antioxidant and enzyme inhibitory activities
Source: BMC Complement Med Ther. 2026 Sep 26;26:279. doi: 10.1186/s12906-026-05588-9 (PMC13616126; doi:10.1186/s12906-026-05588-9)
Supplement: Supplementary file 1 [file 12906_2026_5588_MOESM1_ESM.docx]

**Supplementary Materials**

**Ethnopharmacological insights into type 2 diabetes management by traditional Moroccan herbal teas: HPLC–MS characterization of phenolic compounds and their related antioxidant and enzyme inhibitory activities**

# **Supplementary Figures**

**Suppl. Figure S1.** HPLC-MS chromatogram of *Glycyrrhiza glabra* L. (GG) infusion showing major identified phenolic compounds.

**Suppl. Figure S2.** HPLC-MS chromatogram of *Pelargonium graveolens L.* (PG) infusion showing major identified phenolic compounds.

**Suppl. Figure S3.** HPLC-MS chromatogram of *Rosmarinus officinalis* L. (RO) infusion showing major identified phenolic compounds.

**Suppl. Figure S4.** HPLC-MS chromatogram of *Illicium verum* Hook.f. (BD) infusion showing major identified phenolic compounds.

**Suppl. Figure S5.** HPLC-MS chromatogram of *Matricaria chamomilla* L. (MC) infusion showing major identified phenolic compounds.

**Suppl. Figure S6.** HPLC-MS chromatogram of *Artemisia absinthium* L. (AA) infusion showing major identified phenolic compounds

**Suppl. Figure S7.** HPLC-MS chromatogram of *Mentha pulegium* L.  (MP) infusion showing major identified phenolic compounds.

**Suppl. Figure S8.** HPLC-MS chromatogram of *Ammodaucus leucotrichus* Coss. & Dur.  (AL) infusion showing major identified phenolic compounds.

**Suppl. Figure S9.** HPLC-MS chromatogram of *Hibiscus sabdariffa* L. (HS) infusion showing major identified phenolic compounds.

**Suppl. Figure S10.** HPLC-MS chromatogram of *Lippia citriodora* Kunth.  (LC) infusion showing major identified phenolic compounds.

**Suppl. Figure S11.** HPLC-MS chromatogram of *Calamintha officinalis* Moench.  (CO) infusion showing major identified phenolic compounds.

**Suppl. Figure S12.** HPLC-MS chromatogram of *Origanum majorana* L.  (OM) infusion showing major identified phenolic compounds.

**Suppl. Figure S13.** HPLC-MS chromatogram of *Mentha viridis* L.  (MV) infusion showing major identified phenolic compounds.

**Suppl. Figure S14.** HPLC-MS chromatogram of *Mentha x piperita* L.  (NA) infusion showing major identified phenolic compounds.

**Suppl. Figure S15.** HPLC-MS chromatogram of *Foeniculum vulgare* Mill.  (FV) infusion showing major identified phenolic compounds.

**Suppl. Figure S16.** HPLC-MS chromatogram of *Myrtus communis* L.  (RI) infusion showing major identified phenolic compounds.

**Suppl. Figure S17.** HPLC-MS chromatogram of *Salvia officinalis* L.  (SO) infusion showing major identified phenolic compounds.

**Suppl. Figure S18.** HPLC-MS chromatogram of *Mentha rotundifolia* L.  (MR) infusion showing major identified phenolic compounds.

**Suppl. Figure S19.** HPLC-MS chromatogram of *Origanum compactum* Benth.  (OC) infusion showing major identified phenolic compounds.

**Suppl. Figure S20.** HPLC-MS chromatogram of *Thymus broussonetti* Boiss.  (TB) infusion showing major identified phenolic compounds.

**Suppl. Figure S21.** HPLC-MS chromatogram of *Citrus aurantium* L.  (CA) infusion showing major identified phenolic compounds.

# **Supplementary Tables**

**Suppl. Table S1**: External calibration equations and correlation coefficients (R^2^) of the analytical standards used for HPLC-MS quantification of phenolic compounds. Linearity across the range of 0.1–100 µg mL^-1^

| Compound | Class | Linear equation (Peak area = m⋅C+c) | Correlation coefficient (R^2^) |
| --- | --- | --- | --- |
| Phenolic acids and their derivatives | | | |
| Pyrogallol | Simple Phenol | y=13513x-516,05 | 0.9999 |
| Gallic acid | Hydroxybenzoic acid | y=3469,8x+238,44 | 0.9997 |
| Chlorogenic acid | Hydroxycinnamic acid | y=1926,3x+1740,6 | 0.999 |
| Pyrocatechol | Simple Phenol | y=6816,9x+3231,1 | 0.9938 |
| 4-hydroxybenzoic acid | Hydroxybenzoic acid | y=4914,7x+3190,7 | 0.9996 |
| Caffeic acid | Hydroxycinnamic acid | y=1639,6x+1323,6 | 0.999 |
| Syringic acid | Hydroxybenzoic acid | y=5584,7x+3138,4 | 0.9996 |
| Vanillic acid | Hydroxybenzoic acid | y=215,22x+228,33 | 0.9993 |
| 4-Hydroxycinnamic acid (*p*-Coumaric acid) | Hydroxycinnamic acid | y=9762,1x+21139 | 0.9987 |
| Sinapic acid | Hydroxycinnamic acid | y=11620x+35989 | 0.9946 |
| Ferulic acid | Hydroxycinnamic acid | y=2394,6x+8476,6 | 0.9967 |
| Salicylic acid | Hydroxybenzoic acid | y=22864x+18854 | 0.9997 |
| Rosmarinic acid | Dimer Ester | y=7433,7x+14043 | 0.9979 |
| Resveratrol | Stilbene | y=6,520x+8,500 | 0.9949 |
| Tannic acid | Tannin | y=17613x+7282,6 | 0.9949 |
| Flavonoids | | | |
| Catechin | Flavan-3-ol | y=1639,6x+1323,6 | 0.9996 |
| Epicatechin | Flavan-3-ol | y=3336,3x-439,37 | 0.9942 |
| Rutin | Flavonol glycoside | y=10823x+946,86 | 0.9998 |
| Quercetin | Flavonol aglycone | y=47197x+10555 | 0.9999 |
| y = Peak Area (Arbitrary units)  x = Concentration (µg.mL^-1^)  m = Slope (Sensitivity)  c = y-intercept (Background signal) | | | |

**Suppl. Table S2**: Significant difference of DPPH assay results between extracts was tested using one way ANOVA followed by post HOC test at *p*<0.05

| group 1 | group 2 | p-value |
| --- | --- | --- |
| GG | PG | 1 |
| GG | RO | 0.938392 |
| GG | BD | 0.98711 |
| GG | MC | 1 |
| GG | AA | 0.999999 |
| GG | MP | 2.73E-14 |
| GG | AL | 0.983781 |
| GG | HS | 0.999994 |
| GG | LC | 1 |
| GG | CO | 2.73E-14 |
| GG | OM | 5.42E-10 |
| GG | MV | 0.999999 |
| GG | NA | 2.73E-14 |
| GG | FV | 0.999999 |
| GG | RI | 0.210801 |
| GG | SO | 0.00701 |
| GG | MR | 2.73E-14 |
| GG | OC | 6.5E-05 |
| GG | TB | 2.73E-14 |
| GG | CA | 7.8E-11 |
| PG | RO | 0.938392 |
| PG | BD | 0.98711 |
| PG | MC | 1 |
| PG | AA | 0.999999 |
| PG | MP | 2.73E-14 |
| PG | AL | 0.983781 |
| PG | HS | 0.999994 |
| PG | LC | 1 |
| PG | CO | 2.73E-14 |
| PG | OM | 5.42E-10 |
| PG | MV | 0.999999 |
| PG | NA | 2.73E-14 |
| PG | FV | 0.999999 |
| PG | RI | 0.210801 |
| PG | SO | 0.00701 |
| PG | MR | 2.73E-14 |
| PG | OC | 6.5E-05 |
| PG | TB | 2.73E-14 |
| PG | CA | 7.8E-11 |
| RO | BD | 0.115824 |
| RO | MC | 0.878563 |
| RO | AA | 0.49631 |
| RO | MP | 2.73E-14 |
| RO | AL | 0.164724 |
| RO | HS | 0.421951 |
| RO | LC | 0.996421 |
| RO | CO | 3.18E-14 |
| RO | OM | 1.49E-07 |
| RO | MV | 0.49631 |
| RO | NA | 2.73E-14 |
| RO | FV | 0.999911 |
| RO | RI | 0.998734 |
| RO | SO | 0.49631 |
| RO | MR | 2.73E-14 |
| RO | OC | 0.018215 |
| RO | TB | 2.73E-14 |
| RO | CA | 1.85E-08 |
| BD | MC | 0.996421 |
| BD | AA | 0.999999 |
| BD | MP | 2.73E-14 |
| BD | AL | 1 |
| BD | HS | 1 |
| BD | LC | 0.878563 |
| BD | CO | 2.73E-14 |
| BD | OM | 6.46E-12 |
| BD | MV | 0.999999 |
| BD | NA | 2.73E-14 |
| BD | FV | 0.688866 |
| BD | RI | 0.003619 |
| BD | SO | 5.43E-05 |
| BD | MR | 2.73E-14 |
| BD | OC | 4.3E-07 |
| BD | TB | 2.73E-14 |
| BD | CA | 1.09E-12 |
| MC | AA | 1 |
| MC | MP | 2.73E-14 |
| MC | AL | 0.994478 |
| MC | HS | 1 |
| MC | LC | 1 |
| MC | CO | 2.73E-14 |
| MC | OM | 3.32E-10 |
| MC | MV | 1 |
| MC | NA | 2.73E-14 |
| MC | FV | 0.999984 |
| MC | RI | 0.14852 |
| MC | SO | 0.004276 |
| MC | MR | 2.73E-14 |
| MC | OC | 3.79E-05 |
| MC | TB | 2.73E-14 |
| MC | CA | 4.85E-11 |
| AA | MP | 2.73E-14 |
| AA | AL | 0.999993 |
| AA | HS | 1 |
| AA | LC | 0.999295 |
| AA | CO | 2.73E-14 |
| AA | OM | 5.68E-11 |
| AA | MV | 1 |
| AA | NA | 2.73E-14 |
| AA | FV | 0.98711 |
| AA | RI | 0.033388 |
| AA | SO | 0.000652 |
| AA | MR | 2.73E-14 |
| AA | OC | 5.25E-06 |
| AA | TB | 2.73E-14 |
| AA | CA | 8.76E-12 |
| MP | AL | 2.73E-14 |
| MP | HS | 2.73E-14 |
| MP | LC | 2.73E-14 |
| MP | CO | 0.210801 |
| MP | OM | 1.46E-09 |
| MP | MV | 2.73E-14 |
| MP | NA | 0.00027 |
| MP | FV | 2.73E-14 |
| MP | RI | 2.73E-14 |
| MP | SO | 2.86E-14 |
| MP | MR | 0.999999 |
| MP | OC | 7.9E-14 |
| MP | TB | 0.999911 |
| MP | CA | 1.11E-08 |
| AL | HS | 0.999999 |
| AL | LC | 0.883897 |
| AL | CO | 2.75E-14 |
| AL | OM | 1.03E-10 |
| AL | MV | 0.999993 |
| AL | NA | 2.73E-14 |
| AL | FV | 0.720311 |
| AL | RI | 0.008643 |
| AL | SO | 0.00022 |
| AL | MR | 2.73E-14 |
| AL | OC | 2.89E-06 |
| AL | TB | 2.73E-14 |
| AL | CA | 1.9E-11 |
| HS | LC | 0.997827 |
| HS | CO | 2.73E-14 |
| HS | OM | 4.14E-11 |
| HS | MV | 1 |
| HS | NA | 2.73E-14 |
| HS | FV | 0.974022 |
| HS | RI | 0.024746 |
| HS | SO | 0.000459 |
| HS | MR | 2.73E-14 |
| HS | OC | 3.66E-06 |
| HS | TB | 2.73E-14 |
| HS | CA | 6.46E-12 |
| LC | CO | 2.75E-14 |
| LC | OM | 2.04E-09 |
| LC | MV | 0.999295 |
| LC | NA | 2.73E-14 |
| LC | FV | 1 |
| LC | RI | 0.458606 |
| LC | SO | 0.024746 |
| LC | MR | 2.73E-14 |
| LC | OC | 0.00027 |
| LC | TB | 2.73E-14 |
| LC | CA | 2.82E-10 |
| CO | OM | 2.65E-05 |
| CO | MV | 2.73E-14 |
| CO | NA | 1.31E-08 |
| CO | FV | 2.78E-14 |
| CO | RI | 1.32E-13 |
| CO | SO | 3.53E-12 |
| CO | MR | 0.074039 |
| CO | OC | 2.4E-10 |
| CO | TB | 0.01559 |
| CO | CA | 0.000226 |
| OM | MV | 5.68E-11 |
| OM | NA | 2.8E-14 |
| OM | FV | 5.6E-09 |
| OM | RI | 9E-06 |
| OM | SO | 0.000652 |
| OM | MR | 2.59E-09 |
| OM | OC | 0.051579 |
| OM | TB | 6.65E-11 |
| OM | CA | 1 |
| MV | NA | 2.73E-14 |
| MV | FV | 0.98711 |
| MV | RI | 0.033388 |
| MV | SO | 0.000652 |
| MV | MR | 2.73E-14 |
| MV | OC | 5.25E-06 |
| MV | TB | 2.73E-14 |
| MV | CA | 8.76E-12 |
| NA | FV | 2.73E-14 |
| NA | RI | 2.73E-14 |
| NA | SO | 2.73E-14 |
| NA | MR | 0.017557 |
| NA | OC | 2.73E-14 |
| NA | TB | 0.00701 |
| NA | CA | 3.06E-14 |
| FV | RI | 0.688866 |
| FV | SO | 0.059376 |
| FV | MR | 2.73E-14 |
| FV | OC | 0.000776 |
| FV | TB | 2.73E-14 |
| FV | CA | 7.53E-10 |
| RI | SO | 0.997827 |
| RI | MR | 2.78E-14 |
| RI | OC | 0.352774 |
| RI | TB | 2.73E-14 |
| RI | CA | 1.05E-06 |
| SO | MR | 3.55E-14 |
| SO | OC | 0.991308 |
| SO | TB | 2.75E-14 |
| SO | CA | 7.77E-05 |
| MR | OC | 2.57E-13 |
| MR | TB | 1 |
| MR | CA | 1.6E-08 |
| OC | TB | 3.11E-14 |
| OC | CA | 0.008246 |
| TB | CA | 4.6E-10 |

**Suppl. Table S3**: Significant difference of ABTS assay results between extracts was tested using one-way ANOVA followed by post HOC test at *p*<0.05

| group 1 | group 2 | p-value |
| --- | --- | --- |
| GG | PG | 1 |
| GG | RO | 0.999992 |
| GG | BD | 0.999956 |
| GG | MC | 1 |
| GG | AA | 0.447175 |
| GG | MP | 1.71E-05 |
| GG | AL | 0.999952 |
| GG | HS | 0.112238 |
| GG | LC | 0.999579 |
| GG | CO | 0.001303 |
| GG | OM | 1.69E-06 |
| GG | MV | 0.999999 |
| GG | NA | 4.89E-13 |
| GG | FV | 1 |
| GG | RI | 0.698921 |
| GG | SO | 0.366279 |
| GG | MR | 2.71E-05 |
| GG | OC | 0.080441 |
| GG | TB | 1.03E-08 |
| GG | CA | 4.26E-05 |
| PG | RO | 1 |
| PG | BD | 0.986852 |
| PG | MC | 1 |
| PG | AA | 0.535362 |
| PG | MP | 6.19E-06 |
| PG | AL | 0.991536 |
| PG | HS | 0.013557 |
| PG | LC | 0.999995 |
| PG | CO | 0.000795 |
| PG | OM | 4.76E-07 |
| PG | MV | 1 |
| PG | NA | 6.86E-14 |
| PG | FV | 1 |
| PG | RI | 0.801131 |
| PG | SO | 0.440396 |
| PG | MR | 1.39E-05 |
| PG | OC | 0.082848 |
| PG | TB | 1.79E-09 |
| PG | CA | 1.71E-05 |
| RO | BD | 0.818535 |
| RO | MC | 0.998788 |
| RO | AA | 0.854118 |
| RO | MP | 3.49E-05 |
| RO | AL | 0.878025 |
| RO | HS | 0.002702 |
| RO | LC | 1 |
| RO | CO | 0.004235 |
| RO | OM | 2.61E-06 |
| RO | MV | 1 |
| RO | NA | 1.68E-13 |
| RO | FV | 0.999999 |
| RO | RI | 0.976494 |
| RO | SO | 0.778105 |
| RO | MR | 6.56E-05 |
| RO | OC | 0.273665 |
| RO | TB | 8.63E-09 |
| RO | CA | 9.68E-05 |
| BD | MC | 0.999996 |
| BD | AA | 0.031417 |
| BD | MP | 6.14E-08 |
| BD | AL | 1 |
| BD | HS | 0.434631 |
| BD | LC | 0.626158 |
| BD | CO | 7.1E-06 |
| BD | OM | 5.43E-09 |
| BD | MV | 0.875749 |
| BD | NA | 3.06E-14 |
| BD | FV | 0.998055 |
| BD | RI | 0.085846 |
| BD | SO | 0.02164 |
| BD | MR | 2.16E-07 |
| BD | OC | 0.001187 |
| BD | TB | 3E-11 |
| BD | CA | 1.62E-07 |
| MC | AA | 0.187645 |
| MC | MP | 7.55E-07 |
| MC | AL | 0.999995 |
| MC | HS | 0.081518 |
| MC | LC | 0.984912 |
| MC | CO | 9.58E-05 |
| MC | OM | 6.14E-08 |
| MC | MV | 0.999669 |
| MC | NA | 3.79E-14 |
| MC | FV | 1 |
| MC | RI | 0.391663 |
| MC | SO | 0.139449 |
| MC | MR | 2.09E-06 |
| MC | OC | 0.013816 |
| MC | TB | 2.73E-10 |
| MC | CA | 2.06E-06 |
| AA | MP | 0.082209 |
| AA | AL | 0.059213 |
| AA | HS | 2.59E-05 |
| AA | LC | 0.951911 |
| AA | CO | 0.813052 |
| AA | OM | 0.011454 |
| AA | MV | 0.7994 |
| AA | NA | 7.3E-10 |
| AA | FV | 0.395465 |
| AA | RI | 1 |
| AA | SO | 1 |
| AA | MR | 0.063844 |
| AA | OC | 1 |
| AA | TB | 6.44E-05 |
| AA | CA | 0.160057 |
| MP | AL | 6.15E-07 |
| MP | HS | 3.86E-11 |
| MP | LC | 8.9E-05 |
| MP | CO | 0.982373 |
| MP | OM | 0.999996 |
| MP | MV | 2.44E-05 |
| MP | NA | 2.23E-06 |
| MP | FV | 3.02E-06 |
| MP | RI | 0.029944 |
| MP | SO | 0.113799 |
| MP | MR | 1 |
| MP | OC | 0.152331 |
| MP | TB | 0.372342 |
| MP | CA | 1 |
| AL | HS | 0.697138 |
| AL | LC | 0.729008 |
| AL | CO | 4.63E-05 |
| AL | OM | 6.52E-08 |
| AL | MV | 0.918421 |
| AL | NA | 6.76E-14 |
| AL | FV | 0.998647 |
| AL | RI | 0.139517 |
| AL | SO | 0.042931 |
| AL | MR | 1.29E-06 |
| AL | OC | 0.004284 |
| AL | TB | 4.99E-10 |
| AL | CA | 1.5E-06 |
| HS | LC | 0.001094 |
| HS | CO | 2.66E-09 |
| HS | OM | 4.64E-12 |
| HS | MV | 0.003796 |
| HS | NA | 2.91E-14 |
| HS | FV | 0.025691 |
| HS | RI | 8.95E-05 |
| HS | SO | 1.68E-05 |
| HS | MR | 2.47E-10 |
| HS | OC | 3.3E-07 |
| HS | TB | 7.87E-14 |
| HS | CA | 9.1E-11 |
| LC | CO | 0.010094 |
| LC | OM | 6.59E-06 |
| LC | MV | 1 |
| LC | NA | 3.08E-13 |
| LC | FV | 0.999793 |
| LC | RI | 0.996783 |
| LC | SO | 0.909817 |
| LC | MR | 0.000151 |
| LC | OC | 0.452552 |
| LC | TB | 2.05E-08 |
| LC | CA | 0.000246 |
| CO | OM | 0.609593 |
| CO | MV | 0.003017 |
| CO | NA | 2.05E-08 |
| CO | FV | 0.000389 |
| CO | RI | 0.550182 |
| CO | SO | 0.881991 |
| CO | MR | 0.921506 |
| CO | OC | 0.96996 |
| CO | TB | 0.009082 |
| CO | CA | 0.99883 |
| OM | MV | 1.83E-06 |
| OM | NA | 2.98E-05 |
| OM | FV | 2.36E-07 |
| OM | RI | 0.003618 |
| OM | SO | 0.016882 |
| OM | MR | 1 |
| OM | OC | 0.018985 |
| OM | TB | 0.895818 |
| OM | CA | 0.999484 |
| MV | NA | 1.36E-13 |
| MV | FV | 1 |
| MV | RI | 0.958143 |
| MV | SO | 0.713208 |
| MV | MR | 4.77E-05 |
| MV | OC | 0.219417 |
| MV | TB | 6.21E-09 |
| MV | CA | 6.77E-05 |
| NA | FV | 5.25E-14 |
| NA | RI | 2.47E-10 |
| NA | SO | 1.07E-09 |
| NA | MR | 8.95E-05 |
| NA | OC | 2.02E-10 |
| NA | TB | 0.010094 |
| NA | CA | 8.2E-07 |
| FV | RI | 0.668466 |
| FV | SO | 0.31294 |
| FV | MR | 7.3E-06 |
| FV | OC | 0.046514 |
| FV | TB | 9.36E-10 |
| FV | CA | 8.3E-06 |
| RI | SO | 1 |
| RI | MR | 0.02489 |
| RI | OC | 0.999933 |
| RI | TB | 1.86E-05 |
| RI | CA | 0.063551 |
| SO | MR | 0.086844 |
| SO | OC | 1 |
| SO | TB | 9.92E-05 |
| SO | CA | 0.213505 |
| MR | OC | 0.120222 |
| MR | TB | 0.851148 |
| MR | CA | 1 |
| OC | TB | 6.03E-05 |
| OC | CA | 0.292997 |
| TB | CA | 0.203651 |

**Suppl. Table S4**: Significant difference of FRAP assay results between extracts was tested using one-way ANOVA followed by post HOC test at *p*<0.05

| group 1 | group 2 | p-value |
| --- | --- | --- |
| GG | PG | 0.012142 |
| GG | RO | 1.06E-06 |
| GG | BD | 6.62E-08 |
| GG | MC | 5.09E-11 |
| GG | AA | 5.46E-09 |
| GG | MP | 2.5E-14 |
| GG | AL | 1.86E-06 |
| GG | HS | 3.65E-06 |
| GG | LC | 6.48E-14 |
| GG | CO | 2.55E-14 |
| GG | OM | 2.5E-14 |
| GG | MV | 0.167789 |
| GG | NA | 2.5E-14 |
| GG | FV | 6.06E-07 |
| GG | RI | 1.14E-08 |
| GG | SO | 1.25E-13 |
| GG | MR | 1 |
| GG | OC | 0.928321 |
| GG | TB | 2.5E-14 |
| GG | CA | 2.5E-14 |
| PG | RO | 0.263595 |
| PG | BD | 4.28E-13 |
| PG | MC | 2.61E-14 |
| PG | AA | 0.003319 |
| PG | MP | 2.5E-14 |
| PG | AL | 6.52E-12 |
| PG | HS | 3.93E-11 |
| PG | LC | 3.92E-09 |
| PG | CO | 3.91E-12 |
| PG | OM | 2.5E-14 |
| PG | MV | 0.999926 |
| PG | NA | 2.5E-14 |
| PG | FV | 0.185582 |
| PG | RI | 0.006834 |
| PG | SO | 1.22E-08 |
| PG | MR | 0.015591 |
| PG | OC | 3.91E-05 |
| PG | TB | 2.5E-14 |
| PG | CA | 2.5E-14 |
| RO | BD | 2.54E-14 |
| RO | MC | 2.5E-14 |
| RO | AA | 0.966475 |
| RO | MP | 2.5E-14 |
| RO | AL | 2.89E-14 |
| RO | HS | 6.26E-14 |
| RO | LC | 4.51E-05 |
| RO | CO | 7.66E-09 |
| RO | OM | 2.5E-14 |
| RO | MV | 0.022754 |
| RO | NA | 2.5E-14 |
| RO | FV | 1 |
| RO | RI | 0.993185 |
| RO | SO | 0.00015 |
| RO | MR | 1.41E-06 |
| RO | OC | 3.44E-09 |
| RO | TB | 2.5E-14 |
| RO | CA | 2.5E-14 |
| BD | MC | 0.613318 |
| BD | AA | 2.5E-14 |
| BD | MP | 2.5E-14 |
| BD | AL | 0.999894 |
| BD | HS | 1 |
| BD | LC | 2.5E-14 |
| BD | CO | 2.5E-14 |
| BD | OM | 2.5E-14 |
| BD | MV | 6.08E-12 |
| BD | NA | 2.5E-14 |
| BD | FV | 2.53E-14 |
| BD | RI | 2.51E-14 |
| BD | SO | 2.5E-14 |
| BD | MR | 5.04E-08 |
| BD | OC | 2.38E-05 |
| BD | TB | 2.5E-14 |
| BD | CA | 2.5E-14 |
| MC | AA | 2.5E-14 |
| MC | MP | 2.5E-14 |
| MC | AL | 0.088328 |
| MC | HS | 0.502541 |
| MC | LC | 2.5E-14 |
| MC | CO | 2.5E-14 |
| MC | OM | 2.5E-14 |
| MC | MV | 3.81E-14 |
| MC | NA | 2.5E-14 |
| MC | FV | 2.5E-14 |
| MC | RI | 2.5E-14 |
| MC | SO | 2.5E-14 |
| MC | MR | 3.98E-11 |
| MC | OC | 1.14E-08 |
| MC | TB | 2.5E-14 |
| MC | CA | 2.5E-14 |
| AA | MP | 2.5E-14 |
| AA | AL | 2.52E-14 |
| AA | HS | 2.61E-14 |
| AA | LC | 0.00884 |
| AA | CO | 8.58E-07 |
| AA | OM | 2.5E-14 |
| AA | MV | 0.000132 |
| AA | NA | 2.5E-14 |
| AA | FV | 0.988869 |
| AA | RI | 1 |
| AA | SO | 0.025413 |
| AA | MR | 7.13E-09 |
| AA | OC | 2.6E-11 |
| AA | TB | 2.5E-14 |
| AA | CA | 2.5E-14 |
| MP | AL | 2.5E-14 |
| MP | HS | 2.5E-14 |
| MP | LC | 7.46E-14 |
| MP | CO | 5.26E-09 |
| MP | OM | 0.994856 |
| MP | MV | 2.5E-14 |
| MP | NA | 0.875187 |
| MP | FV | 2.5E-14 |
| MP | RI | 2.5E-14 |
| MP | SO | 4.69E-14 |
| MP | MR | 2.5E-14 |
| MP | OC | 2.5E-14 |
| MP | TB | 0.999999 |
| MP | CA | 0.930866 |
| AL | HS | 1 |
| AL | LC | 2.5E-14 |
| AL | CO | 2.5E-14 |
| AL | OM | 2.5E-14 |
| AL | MV | 1.13E-10 |
| AL | NA | 2.5E-14 |
| AL | FV | 2.74E-14 |
| AL | RI | 2.53E-14 |
| AL | SO | 2.5E-14 |
| AL | MR | 1.41E-06 |
| AL | OC | 0.000696 |
| AL | TB | 2.5E-14 |
| AL | CA | 2.5E-14 |
| HS | LC | 2.5E-14 |
| HS | CO | 2.5E-14 |
| HS | OM | 2.5E-14 |
| HS | MV | 5.45E-10 |
| HS | NA | 2.5E-14 |
| HS | FV | 5.07E-14 |
| HS | RI | 2.66E-14 |
| HS | SO | 2.5E-14 |
| HS | MR | 2.84E-06 |
| HS | OC | 0.000729 |
| HS | TB | 2.5E-14 |
| HS | CA | 2.5E-14 |
| LC | CO | 0.109604 |
| LC | OM | 2.51E-14 |
| LC | MV | 1.91E-10 |
| LC | NA | 2.51E-14 |
| LC | FV | 7.94E-05 |
| LC | RI | 0.004325 |
| LC | SO | 1 |
| LC | MR | 7.44E-14 |
| LC | OC | 2.56E-14 |
| LC | TB | 2.52E-14 |
| LC | CA | 1.99E-13 |
| CO | OM | 7.98E-12 |
| CO | MV | 3.74E-13 |
| CO | NA | 1.75E-12 |
| CO | FV | 1.24E-08 |
| CO | RI | 4.31E-07 |
| CO | SO | 0.048207 |
| CO | MR | 2.56E-14 |
| CO | OC | 2.51E-14 |
| CO | TB | 4.39E-11 |
| CO | CA | 1.1E-07 |
| OM | MV | 2.5E-14 |
| OM | NA | 1 |
| OM | FV | 2.5E-14 |
| OM | RI | 2.5E-14 |
| OM | SO | 2.52E-14 |
| OM | MR | 2.5E-14 |
| OM | OC | 2.5E-14 |
| OM | TB | 1 |
| OM | CA | 0.058833 |
| MV | NA | 2.5E-14 |
| MV | FV | 0.013909 |
| MV | RI | 0.000285 |
| MV | SO | 5.62E-10 |
| MV | MR | 0.201729 |
| MV | OC | 0.00104 |
| MV | TB | 2.5E-14 |
| MV | CA | 2.5E-14 |
| NA | FV | 2.5E-14 |
| NA | RI | 2.5E-14 |
| NA | SO | 2.5E-14 |
| NA | MR | 2.5E-14 |
| NA | OC | 2.5E-14 |
| NA | TB | 0.997565 |
| NA | CA | 0.010367 |
| FV | RI | 0.998567 |
| FV | SO | 0.000263 |
| FV | MR | 8.02E-07 |
| FV | OC | 2.03E-09 |
| FV | TB | 2.5E-14 |
| FV | CA | 2.5E-14 |
| RI | SO | 0.012929 |
| RI | MR | 1.49E-08 |
| RI | OC | 5.09E-11 |
| RI | TB | 2.5E-14 |
| RI | CA | 2.5E-14 |
| SO | MR | 1.5E-13 |
| SO | OC | 2.62E-14 |
| SO | TB | 2.51E-14 |
| SO | CA | 9.36E-14 |
| MR | OC | 0.896923 |
| MR | TB | 2.5E-14 |
| MR | CA | 2.5E-14 |
| OC | TB | 2.5E-14 |
| OC | CA | 2.5E-14 |
| TB | CA | 0.274733 |

**Suppl. Table S5**: HPLC-ESI-MS characterization data of phenolic acids and related compounds: molecular formula, molecular weight, retention time (RT), and major deprotonated ion ([M-H]-) mass-to-charge ratio (*m/z*)

| Phenolic acids | Molecular formula | Molecular weight (Mw) | HPLC-ESI-MS (*m/z*) | |
| --- | --- | --- | --- | --- |
|  |  |  | **RT (min)** | **[M ̶ H] ^̶^** |
| Pyrogallol | C_6_H_6_O_3_ | 126.111 | 0.6 | 125.024 |
| Gallic acid | C_7_H_6_O_5_ | 170.022 | 0.76 | 168.90 |
| Chlorogenic acid | C_16_H_18_O_9_ | 354.311 | 0.83 | 353.202 |
| Pyrocatechol | C_6_H_6_O_2_ | 110.112 | 1.02 | 109.028 |
| 4-hydroxybenzoic acid | C_7_H_6_O_3_ | 138.122 | 1.3 | 137.050 |
| Catechin | C_15_H_14_O_6_ | 290.271 | 1.63 | 289.064 |
| Caffeic acid | C_9_H_8_O_4_ | 180.159 | 1.68 | 179.035 |
| Epicatechin | C_15_H_14_O_6_ | 290.271 | 1.71 | 289.064 |
| Syringic acid | C_9_H_10_O_5_ | 198.174 | 2.05 | 197.045 |
| Vanillic acid | C_8_H_8_O_4_ | 168.148 | 2.12 | 167.036 |
| 4-Hydroxycinnamic acid (*p*-Coumaric acid) | C_9_H_8_O_3_ | 164.160 | 2.21 | 163.042 |
| Rutin | C_27_H_30_O_16_ | 610.153 | 2.63 | 609.1 |
| Sinapic acid | C_11_H_12_O_5_ | 224.212 | 2.74 | 223.061 |
| Ferulic acid | C_10_H_10_O_4_ | 194.186 | 3.12 | 193.050 |
| Quercitin | C_15_H_10_O_7_ | 302.238 | 3.48 | 301.000 |
| Salicylic acid | C_7_H_6_O_3_ | 138.122 | 3.68 | 137.025 |
| Rosmarinic acid | C_18_H_16_O_8_ | 360.318 | 4.01 | 359.054 |
| Resveratrol acid | C_14_H_12_O_3_ | 228.247 | 5.83 | 227.072 |
| Tannic acid | C_76_H_52_O_46_ | 1701.206 | 3.36 | 1700.080 |

**Suppl. Table S6**: Significant difference of *α*-amylase inhibition results between extracts was tested using one way ANOVA followed by post HOC test at *p*<0.05

| group 1 | group 2 | p-value |
| --- | --- | --- |
| GG | PG | 0.016153 |
| GG | RO | 1 |
| GG | BD | 1 |
| GG | MC | 0.995511 |
| GG | AA | 0.871771 |
| GG | MP | 0.979707 |
| GG | AL | 0.987929 |
| GG | HS | 1 |
| GG | LC | 0.551395 |
| GG | CO | 0.985746 |
| GG | OM | 0.993846 |
| GG | MV | 0.036657 |
| GG | NA | 1 |
| GG | FV | 0.998435 |
| GG | RI | 1 |
| GG | SO | 0.999998 |
| GG | MR | 0.069875 |
| GG | TB | 1 |
| GG | OC | 0.13991 |
| GG | CA | 0.999353 |
| PG | RO | 0.016259 |
| PG | BD | 0.003051 |
| PG | MC | 0.42343 |
| PG | AA | 2.75E-05 |
| PG | MP | 0.567342 |
| PG | AL | 0.511659 |
| PG | HS | 0.001663 |
| PG | LC | 4.98E-06 |
| PG | CO | 0.528719 |
| PG | OM | 0.000174 |
| PG | MV | 1 |
| PG | NA | 0.045937 |
| PG | FV | 0.000289 |
| PG | RI | 0.11599 |
| PG | SO | 0.1396 |
| PG | MR | 1.5E-07 |
| PG | TB | 0.004166 |
| PG | OC | 0.999998 |
| PG | CA | 0.00038 |
| RO | BD | 1 |
| RO | MC | 0.995596 |
| RO | AA | 0.870727 |
| RO | MP | 0.979994 |
| RO | AL | 0.98812 |
| RO | HS | 1 |
| RO | LC | 0.549774 |
| RO | CO | 0.985964 |
| RO | OM | 0.993734 |
| RO | MV | 0.036884 |
| RO | NA | 1 |
| RO | FV | 0.998399 |
| RO | RI | 1 |
| RO | SO | 0.999998 |
| RO | MR | 0.069475 |
| RO | TB | 1 |
| RO | OC | 0.140632 |
| RO | CA | 0.999336 |
| BD | MC | 0.888303 |
| BD | AA | 0.993891 |
| BD | MP | 0.781457 |
| BD | AL | 0.826287 |
| BD | HS | 1 |
| BD | LC | 0.891769 |
| BD | CO | 0.813006 |
| BD | OM | 0.999993 |
| BD | MV | 0.007489 |
| BD | NA | 0.999985 |
| BD | FV | 1 |
| BD | RI | 0.998201 |
| BD | SO | 0.996231 |
| BD | MR | 0.244766 |
| BD | TB | 1 |
| BD | OC | 0.034958 |
| BD | CA | 1 |
| MC | AA | 0.101824 |
| MC | MP | 1 |
| MC | AL | 1 |
| MC | HS | 0.790052 |
| MC | LC | 0.02725 |
| MC | CO | 1 |
| MC | OM | 0.325651 |
| MC | MV | 0.628433 |
| MC | NA | 0.999934 |
| MC | FV | 0.421257 |
| MC | RI | 1 |
| MC | SO | 1 |
| MC | MR | 0.001147 |
| MC | TB | 0.92579 |
| MC | OC | 0.925313 |
| MC | CA | 0.477947 |
| AA | MP | 0.060912 |
| AA | AL | 0.07431 |
| AA | HS | 0.998959 |
| AA | LC | 1 |
| AA | CO | 0.069934 |
| AA | OM | 1 |
| AA | MV | 7.32E-05 |
| AA | NA | 0.644237 |
| AA | FV | 1 |
| AA | RI | 0.387666 |
| AA | SO | 0.338179 |
| AA | MR | 0.985714 |
| AA | TB | 0.987117 |
| AA | OC | 0.000432 |
| AA | CA | 0.999999 |
| MP | AL | 1 |
| MP | HS | 0.654748 |
| MP | LC | 0.015142 |
| MP | CO | 1 |
| MP | OM | 0.219309 |
| MP | MV | 0.767434 |
| MP | NA | 0.999113 |
| MP | FV | 0.295599 |
| MP | RI | 0.999996 |
| MP | SO | 0.999999 |
| MP | MR | 0.000587 |
| MP | TB | 0.837958 |
| MP | OC | 0.974042 |
| MP | CA | 0.343469 |
| AL | HS | 0.708528 |
| AL | LC | 0.01897 |
| AL | CO | 1 |
| AL | OM | 0.25631 |
| AL | MV | 0.717095 |
| AL | NA | 0.999637 |
| AL | FV | 0.340328 |
| AL | RI | 0.999999 |
| AL | SO | 1 |
| AL | MR | 0.000757 |
| AL | TB | 0.87601 |
| AL | OC | 0.959778 |
| AL | CA | 0.392002 |
| HS | LC | 0.953831 |
| HS | CO | 0.692219 |
| HS | OM | 1 |
| HS | MV | 0.004164 |
| HS | NA | 0.99975 |
| HS | FV | 1 |
| HS | RI | 0.990748 |
| HS | SO | 0.983778 |
| HS | MR | 0.349559 |
| HS | TB | 1 |
| HS | OC | 0.020464 |
| HS | CA | 1 |
| LC | CO | 0.017704 |
| LC | OM | 0.999891 |
| LC | MV | 1.33E-05 |
| LC | NA | 0.305494 |
| LC | FV | 0.99927 |
| LC | RI | 0.143211 |
| LC | SO | 0.119096 |
| LC | MR | 0.999909 |
| LC | TB | 0.845436 |
| LC | OC | 8.05E-05 |
| LC | CA | 0.998258 |
| CO | OM | 0.244487 |
| CO | MV | 0.732973 |
| CO | NA | 0.999516 |
| CO | FV | 0.326156 |
| CO | RI | 0.999999 |
| CO | SO | 1 |
| CO | MR | 0.0007 |
| CO | TB | 0.864899 |
| CO | OC | 0.964667 |
| CO | CA | 0.376705 |
| OM | MV | 0.000456 |
| OM | NA | 0.93733 |
| OM | FV | 1 |
| OM | RI | 0.766001 |
| OM | SO | 0.714158 |
| OM | MR | 0.81333 |
| OM | TB | 0.999963 |
| OM | OC | 0.002545 |
| OM | CA | 1 |
| MV | NA | 0.096489 |
| MV | FV | 0.000751 |
| MV | RI | 0.220344 |
| MV | SO | 0.258514 |
| MV | MR | 3.97E-07 |
| MV | TB | 0.010108 |
| MV | OC | 1 |
| MV | CA | 0.000984 |
| NA | FV | 0.971761 |
| NA | RI | 1 |
| NA | SO | 1 |
| NA | MR | 0.02559 |
| NA | TB | 0.999998 |
| NA | OC | 0.303533 |
| NA | CA | 0.983117 |
| FV | RI | 0.852169 |
| FV | SO | 0.809396 |
| FV | MR | 0.718804 |
| FV | TB | 0.999997 |
| FV | OC | 0.004106 |
| FV | CA | 1 |
| RI | SO | 1 |
| RI | MR | 0.008766 |
| RI | TB | 0.999371 |
| RI | OC | 0.544035 |
| RI | CA | 0.890392 |
| SO | MR | 0.006884 |
| SO | TB | 0.998522 |
| SO | OC | 0.601461 |
| SO | CA | 0.853737 |
| MR | TB | 0.199654 |
| MR | OC | 2.39E-06 |
| MR | CA | 0.661829 |
| TB | OC | 0.045773 |
| TB | CA | 1 |
| OC | CA | 0.005309 |

**Suppl. Table S7**: Significant difference of *α*-glucosidase inhibition results between extracts was tested using one-way ANOVA followed by post HOC test at *p*<0.05

| group 1 | group 2 | p-value |
| --- | --- | --- |
| GG | PG | 0.999998 |
| GG | RO | 1 |
| GG | BD | 0.764214 |
| GG | MC | 0.484943 |
| GG | AA | 0.997746 |
| GG | MP | 0.472448 |
| GG | AL | 0.356899 |
| GG | HS | 0.991657 |
| GG | LC | 0.999999 |
| GG | CO | 0.002404 |
| GG | MO | 1 |
| GG | MV | 0.68861 |
| GG | NA | 0.999999 |
| GG | FV | 0.152738 |
| GG | MC | 0.00235 |
| GG | SO | 0.075204 |
| GG | MR | 0.029913 |
| GG | OC | 0.265771 |
| GG | TB | 4.59E-13 |
| GG | CA | 0.998672 |
| PG | RO | 0.999997 |
| PG | BD | 0.99676 |
| PG | MC | 0.950485 |
| PG | AA | 1 |
| PG | MP | 0.945899 |
| PG | AL | 0.884953 |
| PG | HS | 1 |
| PG | LC | 1 |
| PG | CO | 0.028176 |
| PG | MO | 1 |
| PG | MV | 0.991657 |
| PG | NA | 0.978686 |
| PG | FV | 0.628256 |
| PG | MC | 0.027618 |
| PG | SO | 0.418828 |
| PG | MR | 0.22243 |
| PG | OC | 0.803087 |
| PG | TB | 6.91E-14 |
| PG | CA | 1 |
| RO | BD | 0.752897 |
| RO | MC | 0.472448 |
| RO | AA | 0.997335 |
| RO | MP | 0.460056 |
| RO | AL | 0.345985 |
| RO | HS | 0.990459 |
| RO | LC | 0.999999 |
| RO | CO | 0.002271 |
| RO | MO | 1 |
| RO | MV | 0.676226 |
| RO | NA | 0.999999 |
| RO | FV | 0.146713 |
| RO | MC | 0.00222 |
| RO | SO | 0.071878 |
| RO | MR | 0.028459 |
| RO | OC | 0.256676 |
| RO | TB | 4.82E-13 |
| RO | CA | 0.998409 |
| BD | MC | 1 |
| BD | AA | 0.999995 |
| BD | MP | 1 |
| BD | AL | 1 |
| BD | HS | 1 |
| BD | LC | 0.994698 |
| BD | CO | 0.533158 |
| BD | MO | 0.964013 |
| BD | MV | 1 |
| BD | NA | 0.251321 |
| BD | FV | 0.999931 |
| BD | MC | 0.52804 |
| BD | SO | 0.997668 |
| BD | MR | 0.969342 |
| BD | OC | 0.999999 |
| BD | TB | 2.49E-14 |
| BD | CA | 0.999987 |
| MC | AA | 0.998672 |
| MC | MP | 1 |
| MC | AL | 1 |
| MC | HS | 0.999774 |
| MC | LC | 0.934784 |
| MC | CO | 0.805154 |
| MC | MO | 0.811292 |
| MC | MV | 1 |
| MC | NA | 0.104202 |
| MC | FV | 1 |
| MC | MC | 0.80101 |
| MC | SO | 0.999987 |
| MC | MR | 0.998623 |
| MC | OC | 1 |
| MC | TB | 2.41E-14 |
| MC | CA | 0.997746 |
| AA | MP | 0.998409 |
| AA | AL | 0.992525 |
| AA | HS | 1 |
| AA | LC | 1 |
| AA | CO | 0.102422 |
| AA | MO | 0.999997 |
| AA | MV | 0.999966 |
| AA | NA | 0.809256 |
| AA | FV | 0.912126 |
| AA | MC | 0.100669 |
| AA | SO | 0.761968 |
| AA | MR | 0.520378 |
| AA | OC | 0.97722 |
| AA | TB | 3.56E-14 |
| AA | CA | 1 |
| MP | AL | 1 |
| MP | HS | 0.999716 |
| MP | LC | 0.929252 |
| MP | CO | 0.815331 |
| MP | MO | 0.80101 |
| MP | MV | 1 |
| MP | NA | 0.099801 |
| MP | FV | 1 |
| MP | MC | 0.811292 |
| MP | SO | 0.99999 |
| MP | MR | 0.998855 |
| MP | OC | 1 |
| MP | TB | 2.41E-14 |
| MP | CA | 0.997335 |
| AL | HS | 0.998035 |
| AL | LC | 0.858577 |
| AL | CO | 0.899821 |
| AL | MO | 0.68861 |
| AL | MV | 1 |
| AL | NA | 0.064412 |
| AL | FV | 1 |
| AL | MC | 0.896948 |
| AL | SO | 1 |
| AL | MR | 0.999859 |
| AL | OC | 1 |
| AL | TB | 2.4E-14 |
| AL | CA | 0.988843 |
| HS | LC | 1 |
| HS | CO | 0.146713 |
| HS | MO | 0.999957 |
| HS | MV | 0.999998 |
| HS | NA | 0.712976 |
| HS | FV | 0.957239 |
| HS | MC | 0.144357 |
| HS | SO | 0.849696 |
| HS | MR | 0.630809 |
| HS | OC | 0.992101 |
| HS | TB | 3.16E-14 |
| HS | CA | 1 |
| LC | CO | 0.023987 |
| LC | MO | 1 |
| LC | MV | 0.987335 |
| LC | NA | 0.985316 |
| LC | FV | 0.587179 |
| LC | MC | 0.023506 |
| LC | SO | 0.381543 |
| LC | MR | 0.197488 |
| LC | OC | 0.768682 |
| LC | TB | 7.65E-14 |
| LC | CA | 1 |
| CO | MO | 0.010541 |
| CO | MV | 0.615461 |
| CO | NA | 0.000186 |
| CO | FV | 0.990459 |
| CO | MC | 1 |
| CO | SO | 0.999388 |
| CO | MR | 0.999997 |
| CO | OC | 0.951368 |
| CO | TB | 2.36E-14 |
| CO | CA | 0.089876 |
| MO | MV | 0.935854 |
| MO | NA | 0.998623 |
| MO | FV | 0.390711 |
| MO | MC | 0.010319 |
| MO | SO | 0.225701 |
| MO | MR | 0.104202 |
| MO | OC | 0.574297 |
| MO | TB | 1.38E-13 |
| MO | CA | 0.999999 |
| MV | NA | 0.198983 |
| MV | FV | 0.999989 |
| MV | MC | 0.61033 |
| MV | SO | 0.99928 |
| MV | MR | 0.984956 |
| MV | OC | 1 |
| MV | TB | 2.46E-14 |
| MV | CA | 0.999923 |
| NA | FV | 0.020173 |
| NA | MC | 0.000181 |
| NA | SO | 0.008414 |
| NA | MR | 0.002881 |
| NA | OC | 0.042146 |
| NA | TB | 4.15E-12 |
| NA | CA | 0.83865 |
| FV | MC | 0.989943 |
| FV | SO | 1 |
| FV | MR | 1 |
| FV | OC | 1 |
| FV | TB | 2.36E-14 |
| FV | CA | 0.891051 |
| MC | SO | 0.999336 |
| MC | MR | 0.999996 |
| MC | OC | 0.949591 |
| MC | TB | 2.36E-14 |
| MC | CA | 0.088306 |
| SO | MR | 1 |
| SO | OC | 1 |
| SO | TB | 2.38E-14 |
| SO | CA | 0.727301 |
| MR | OC | 0.999988 |
| MR | TB | 2.36E-14 |
| MR | CA | 0.482436 |
| OC | TB | 2.39E-14 |
| OC | CA | 0.968711 |
| TB | CA | 3.72E-14 |
